# A systematic review of tools designed for teacher proxy-report of children’s physical literacy or constituting elements

**DOI:** 10.1186/s12966-021-01162-3

**Published:** 2021-10-08

**Authors:** Inimfon A. Essiet, Natalie J. Lander, Jo Salmon, Michael J. Duncan, Emma L. J. Eyre, Jiani Ma, Lisa M. Barnett

**Affiliations:** 1 School of Health and Social Development, Deakin University, Geelong, Victoria Australia; 2 Centre for Sport, Exercise and Life Sciences, Coventry University, Coventry, UK; 3 School of Education, Deakin University, Geelong, Victoria Australia; 4 Institute for Physical Activity and Nutrition, School of Exercise and Nutrition Science, Deakin University, Geelong, Victoria Australia; 5 Institute for Physical Activity and Nutrition, School of Health and Social Development, Deakin University, Geelong, Victoria Australia

**Keywords:** Assessment, Measurement, Psychometrics, Physical literacy, Child, COSMIN, Systematic review

## Abstract

**Background:**

Physical literacy (PL) in childhood is essential for a healthy active lifestyle, with teachers playing a critical role in guiding its development. Teachers can assist children to acquire the skills, confidence, and creativity required to perform diverse movements and physical activities. However, to detect and directly intervene on the aspects of children’s PL that are suboptimal, teachers require valid and reliable measures. This systematic review critically evaluates the psychometric properties of teacher proxy-report instruments for assessing one or more of the 30 elements within the four domains (physical, psychological, cognitive, social) of the Australian Physical Literacy Framework (APLF), in children aged 5–12 years. Secondary aims were to: examine alignment of each measure (and relevant items) with the APLF and provide recommendations for teachers in assessing PL.

**Methods:**

Seven electronic databases (Academic Search Complete, CINAHL Complete, Education Source, Global Health, MEDLINE Complete, PsycINFO, and SPORTDiscus) were systematically searched originally in October 2019, with an updated search in April 2021. Eligible studies were peer-reviewed English language publications that sampled a population of children with mean age between 5 and 12 years and focused on developing and evaluating at least one psychometric property of a teacher proxy-report instrument for assessing one or more of the 30 APLF elements. The Preferred Reporting Items for Systematic Reviews and Meta-Analyses (PRISMA) guidance was followed for the conduct and reporting of this review. The methodological quality of included studies and quality of psychometric properties of identified tools were evaluated using the COnsensus-based Standards for the selection of health Measurement INstruments (COSMIN) guidance. Alignment of each measure (and relevant items) with the APLF domains and 30 elements was appraised.

**Results:**

Database searches generated 61,412 citations; reduced to 41 studies that evaluated the psychometric properties of 24 teacher proxy-report tools. Six tools were classified as single domain measures (i.e. assessing a single domain of the APLF), eleven as dual-domain measures, and seven as tri-domain measures. No single tool captured all four domains and 30 elements of the APLF. Tools contained items that aligned with all physical, psychological, and social elements; however, four cognitive elements were not addressed by any measure. No tool was assessed for all nine psychometric properties outlined by COSMIN. Included studies reported a median of 3 out of nine psychometric properties. Most reported psychometric properties were construct validity (*n* = 32; 78% of studies), structural validity (*n* = 26; 63% of studies), and internal consistency (*n* = 25; 61% of studies). There was underreporting of content validity, cross-cultural validity, measurement error, and responsiveness. Psychometric data across tools were mostly indeterminate for construct validity, structural validity, and internal consistency.

**Conclusions:**

There is limited evidence to fully support the use of a specific teacher proxy-report tool in practice. Further psychometric testing and detailed reporting of methodological aspects in future validity and reliability studies is needed. Tools have been designed to assess some elements of the framework. However, no comprehensive teacher proxy-report tool exists to assess all 30 elements of the APLF, demonstrating the need for a new tool. It is our recommendation that such tools be developed and psychometrically tested.

**Trial registration:**

This systematic review was registered in the PROSPERO international prospective register of systematic reviews, with registration number CRD42019130936.

**Supplementary Information:**

The online version contains supplementary material available at 10.1186/s12966-021-01162-3.

## Background

Adequate levels of physical activity during childhood are associated with considerable health benefits (e.g., improvement in physical fitness, academic performance, cognition, and executive functioning) [1–3]. Yet, less than 40% of children in many countries accumulate the levels of physical activity necessary for optimal health [4]. The concept of physical literacy (PL) has been explored in multiple sectors including physical education, sports, recreation, and public health, as a framework to better understand the declining levels of physical activity [5, 6]. Growing empirical evidence has demonstrated that PL, or its components, are associated with adherence to physical activity and sedentary behaviour guidelines [7], increased cardiorespiratory fitness [8], resilience [9], and other health indices (including body composition, blood pressure, health related quality of life) [10] in school-aged children.

Of particular interest when determining PL levels are school-aged children (aged 5–12 years) as literature suggest that childhood is a critical developmental period for the formation of skills and attributes (e.g., motor competence) that underlie lifelong physical activity habits [7, 11]. The school setting has been recognized as a suitable environment that affords children with diverse opportunities that can help foster healthy physically active lifestyles, independent of their culture and socioeconomic status [12]. From this equity perspective, schools are also effective sites for targeted physical activity interventions due to the large amount of time children spend attending schools [13]. Teachers (particularly physical educators) have been identified as key players in guiding children’s PL development [14]. They can support PL education, conceptualized as the “teaching and learning of the skills, knowledge, attitudes, and behaviours that enhance the responsibility for engagement in lifelong active lifestyles” [15]. Teachers are also trained to be sensitive to the needs of each child and have a broad basis for knowing their students as they interact with a large number of different children, and thus have a frame of reference on which to base their judgements [16]. Therefore, teachers may be well suited to identify elements (such as motor competence, motivation and confidence) of a child’s PL [17]. For such identification, valid and reliable PL teacher assessment protocols are required.

Recently, PL scholarship has been directed towards designing assessment tools (both subjective and objective) for different targeted users (including preschoolers, children, youth, teachers, parents). Indeed, assessment is crucial to the planning and evaluation of programs targeted at enhancing PL levels, and could help identify domains of a child’s PL that are suboptimal [18]. As such, following Robinson and Randall [19], an effective PL assessment protocol should address all of its constituting domains (e.g., affective, behavioural, physical, and cognitive). However, few protocols have been designed specifically for use by teachers to evaluate children’s PL [19]. Examples include the PLAY*fun* and *basic* [20]; the CAPL via the Canadian Agility and Movement Skill Assessment (CAMSA) and fitness tests [21]; and the PFL via fitness and movement skills tests [22, 23]. These existing teacher assessment tools largely utilize objective observational approaches (i.e. rely on the teacher observing children perform a series of standardized tasks) [24] rather than teacher proxy-report, and have narrowly focused on the physical domain, thereby neglecting the psychological, social, and cognitive aspects of PL. Comparatively, teacher proxy-report instruments (retrospectively completed questionnaires) have received much less attention despite their suitability for assessing large sample sizes and their minimal manual data entry requirements [25, 26]. Literature has further suggested that teacher proxy-reporting presents a promising avenue to obtain more reliable estimates of a child’s PL, as children under 10 often present with limited cognitive ability to make accurate judgements of their own capabilities [27].

**Fig. 1:**
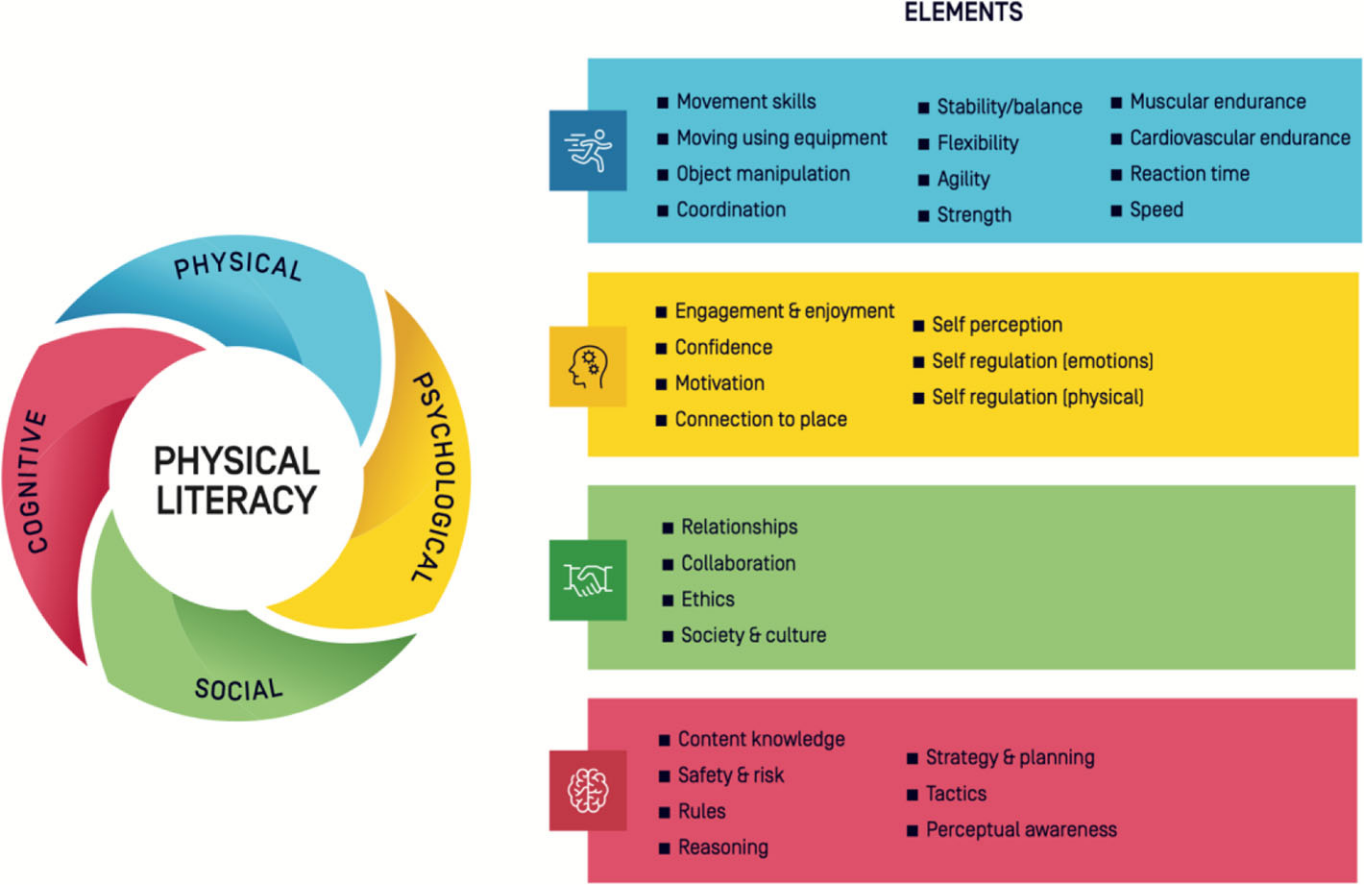
Components of the Australian Physical Literacy Framework adapted with permission from Sport Australia [30])

More specifically, a notable gap in PL assessment is the paucity of teacher proxy-report measures that recognizes components of the expansive and comprehensive Australian Physical Literacy Framework (APLF) [28]. In 2016, after acknowledging the lack of international consensus on PL’s definition, conceptualization, and operationalization, Sport Australia (a Federal Government agency responsible for supporting sport in Australia) proposed arguably the most comprehensive definition and framework for PL to date. See Keegan et al. [29] for a detailed articulation of the Australian definition. The APLF identified a combined total of 30 elements spanning four major domains (physical, psychological, social, and cognitive), as being fundamental to PL development (Fig. 1) [29]. For the purpose of this manuscript, the authors adopt the comprehensive PL definition and framework offered by Sport Australia.

To date, only two systematic reviews have been published in relation to PL assessment [31, 32]. In Edwards et al.’s [31] review, PL assessment/measurement approaches were broadly categorized as qualitative and quantitative. Though quantitative measures for PL and its related constructs were identified, the review did not engage in a detailed and in-depth analysis of the psychometric properties of the measures. Furthermore, the search strategy utilized by authors did not address each individual element (e.g., motivation, confidence, movement skills) of PL, including those belonging to the APLF. More recently, Kaioglou, Venetsanou [32], reviewed existing PL measures used within the context of gymnastics. Like Edwards et al. [31], search terms did not capture individual elements of PL (including APLF elements). Hence, only tools for assessing PL in its entirety were identified (e.g., Canadian Assessment of Physical Literacy [CAPL]; Passport for Life [PFL]; Physical Literacy Assessment for Youth [PLAY]). Both reviews did not focus specifically on identifying teacher proxy-report measures for PL or its constituting elements. Barnett et al. [33] has suggested that teachers have limited guidance when choosing appropriate protocols for assessing PL.

Taking all this into account, the objectives of the current systematic review were two-fold. The primary aim was to critically evaluate the psychometric properties of teacher proxy-report instruments for assessing one or more of the 30 elements within the four domains of the APLF, in children aged 5–12 years. Secondary aims were to examine the alignment of each tool (and relevant items within) with the APLF and provide recommendations for teachers in assessing PL in children aged 5–12 years. A review of this nature will assist teachers (and indeed researchers) in making informed decisions when selecting suitable and psychometrically sound measures for assessing elements within the APLF.

## Methods

### Literature search strategy

The Preferred Reporting Items for Systematic Reviews and Meta-Analyses (PRISMA) [34] and the COnsensus-based Standards for the selection of health Measurement INstruments (COSMIN) guidelines [35–37] were used as methodological and reporting guidelines for this systematic review. See completed PRISMA checklist attached as Additional file 1. Prior to review commencement, details of the review protocol were registered on PROSPERO (CRD42019130936). The first author systematically searched for peer-reviewed articles on seven databases including Academic Search Complete, CINAHL Complete, Education Source, Global Health, MEDLINE Complete, PsycINFO, and SPORTDiscus. These databases encompass areas related to psychology (including psychometrics), education, sport, and health, and were deemed relevant to the comprehensive definition/framework of PL used in this review, and therefore enhanced the likelihood of identifying relevant papers from many diverse disciplines. Date restrictions were not applied to searches. Database searches were originally completed in October 2019 and updated in April 2021. All searches were limited to title, abstract, and keyword. Additional limits of “English language” and “peer review” were applied. To ensure that search terms were not overly simplistic, a comprehensive search filter containing a selection of search terms provided by the COSMIN for finding studies on measurement properties, combined with search terms relevant to the 30 APLF elements (identified from published systematic reviews) were utilized to identify studies concerning the target population (see Additional file 2 for the full search strategy). Reference lists of literature reviews and eligible studies were also searched for additional papers. All searches were performed by the first author with the assistance of the university’s librarian.

### Eligibility criteria

Studies were included if they were: (a) peer-reviewed and written in English Language; (b) study participants included children with mean age between 5 and 12 years; (c) focused on developing and evaluating at least one psychometric property of a teacher proxy-report instrument; and (d) instruments assessed one or more of the 30 elements within the APLF. Because the application of PL goes beyond the context of physical education and encompasses before- and after-school programming, recess, and classroom activities [38, 39] and could be applied in performing arts [40], teacher proxy-report instruments that assessed elements in general contexts (not just in sport and physical activity) were included. For example, instruments assessing “self-regulation” in general, and those assessing self-regulation in the context of physical activity were included.

Studies were excluded if they were: (a) tool manual(s), abstracts (including poster abstracts), conference proceedings, dissertations, commentaries, editorials, review articles, and letters; (b) utilized assessment formats other than teacher proxy-report (e.g., self-report, objective measures); (c) study participants were younger than five and older than 12 years; and (d) utilized proxy-respondents of children not in elementary or primary school, younger than five and older than 12 years. In registering the protocol for this review, it was our initial intention to exclude studies that involved non-typically developing children (such as those with learning difficulties or developmental delay). However, following the literature search, we noted that most teacher proxy-report tools for motor competence (related to the physical domain of PL) were originally designed with the intention of identifying children with developmental coordination disorder (DCD), and in some cases included participants with DCD (for instance, when assessing discriminant validity). As such, these tools were retained in order to ensure motor competence teacher proxy-report measures were not excluded from the review. Measures developed to assess children with other disabilities (i.e. those in relation to elements other than motor competence) were excluded from the review.

### Study selection

Titles and abstracts were exported to Covidence (www.covidence.org), an online software for managing systematic reviews. Following removal of duplicates, the first author screened all titles and abstracts for eligibility, based on the aforementioned criteria. Full text articles were retrieved for further examination where it was not possible to make inclusion decisions based solely on the title and abstract. Following initial selection, full-text articles were independently examined by paired combinations of three review authors (IE - NL, IE - LB, and NL - LB). For consistency, a PICO-based hierarchy of exclusion reasons was developed based on past literature [41], and used to guide the exclusion of studies during the full text review phase (see Additional file 3). Any conflicts between the three reviewers over study inclusion were resolved via review and discussion.

### Data extraction

In line with the criteria proposed by COSMIN, data collection involved extracting information on the general characteristics of included studies as follows: (a) instrument, author(s) and year of publication; (b) general construct assessed; (c) APLF domain(s) assessed; (d) targeted age group/grades; (e) sample population/country; (f) sample size, mean age, standard deviation; (g) instrument available translation; (h) completion time (minutes or seconds); (i) recall period; (j) tool subscale(s)/number of items; (k) response options; (l) psychometric properties evaluated/statistical tests utilized. The data extraction form was piloted on two randomly selected included studies prior to data collection by IE. JM checked all extracted data for completeness and correctness.

### Methodological quality assessment of studies

Following COSMIN’s recommendations, the current review assessed nine measurement properties including: (a) content validity, (b) structural validity, (c) internal consistency, (d) cross-cultural validity, (e) reliability, (f) measurement error, (g) criterion validity, (h) construct validity, and (i) responsiveness – see Prinsen et al. [36] for a definition of each terminology. To evaluate the methodological quality of the selected studies, the recently updated COSMIN Risk of Bias checklist [35, 37] which contains 10 boxes was utilized. Each box of the checklist comprises of 3 to 35 standards for evaluating the statistical design and statistical methods utilized in reliability and validity studies. To date, the COSMIN checklist is the only validated and standardized tool for assessing the methodological quality of health-related outcome measures [42].

Depending on the information reported in each study, items in each box of the checklist were rated on a four-point scale using the descriptors “Very Good”, “Adequate”, “Doubtful”, and “Inadequate”. A “Not Applicable” option was also included for each measurement property. To determine the overall methodological quality for each individual measurement property per study, the lowest rating across the items in the box was taken, a method known as the “the worst score counts” principle. For example, if for a reliability study one item in a box is rated as “Inadequate” despite having all other items rated as “Very Good”, the overall methodological quality of that reliability study will be “Inadequate”. According to COSMIN, this stringent rule is necessary as poor methodological aspects of a study cannot be compensated for by good aspects [37]. To ensure accuracy of the quality assessment, IE completed risk of bias analyses for 22 of the included studies. The articles were then double rated by two independent reviewers (NL, LB) who had both received training on using COSMIN. After disagreements were resolved, IE completed quality assessment for the remaining articles. To summarize the results of methodological quality per tool, authors used a cut-off of ≥60% [43] of measurement properties rated as “Very Good” or “Adequate” across all single studies to indicate “good” methodological quality.

### Quality criteria for measurement properties of single studies and evidence summary

Results obtained from single studies on measurement properties were rated against COSMIN’s updated criteria for good measurement properties. Each result was rated as either sufficient (+), insufficient (−), or indeterminate (?) [36]. For studies reporting on content validity, the quality of the results were rated using the criteria for relevance (5), comprehensiveness (1), and comprehensibility (4) [37]. Regarding hypothesis testing for construct validity and responsiveness, COSMIN recommends setting a priori hypotheses prior to review commencement [35]. Following De Vet et al. [44], for both measurement properties, correlations were expected to be: ≥ 0.50 with instruments measuring similar constructs; < 0.50 and ≥ 0.30 with instruments measuring related but dissimilar constructs; and < 0.30 with instruments measuring unrelated constructs. No hypotheses were formulated for expected differences between groups (e.g., age, gender) for discriminant and known-groups validity.

Due to considerable differences across studies in terms of sample characteristics and size, statistical tests utilized, reliability or validity type investigated, results from single studies could not be pooled in a meta-analysis. Therefore, as recommended by the COSMIN, an overall rating of study results per measurement property per tool was summarized as sufficient (+), insufficient (−), indeterminate (?), or inconsistent (±). Specifically, an overall rating was determined through combining the scoring of each single study; if ≥75% of the studies displayed the same scoring, that scoring became the overall rating (+ or −), whereas if < 75% of studies displayed the same scoring, the overall rating became inconsistent (±) [36].

## Results

### Search results

**Fig. 2:**
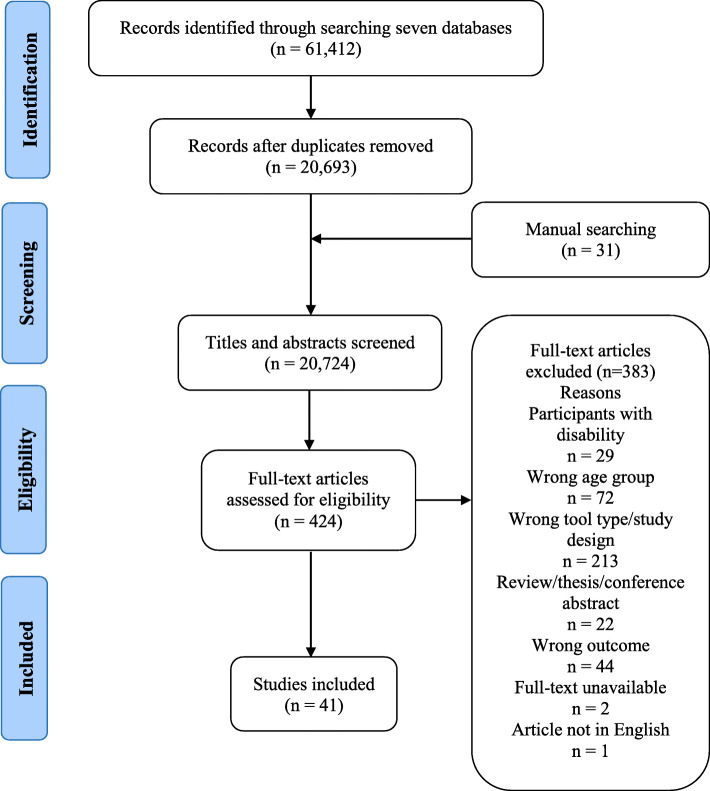
PRISMA flow diagram of the study selection process

Initial searches of the seven databases in October 2019 generated a combined total of 56,615 citations. The updated search in April 2021 identified 4797 new citations. Following removal of duplicates, title and abstract screening of 20,724 references (including an additional 31 articles identified through manual searching), yielded 424 articles deemed potentially relevant. After eligibility criteria were applied to full-text versions of the 424 publications, a total of 41 studies evaluating the psychometric properties of 24 unique teacher proxy-report measures for elements within the APLF were identified. A flow chart of study selection was prepared in accordance to the PRISMA statement (detailed in Fig. 2).

### General characteristics of included studies

**Fig. 3:**
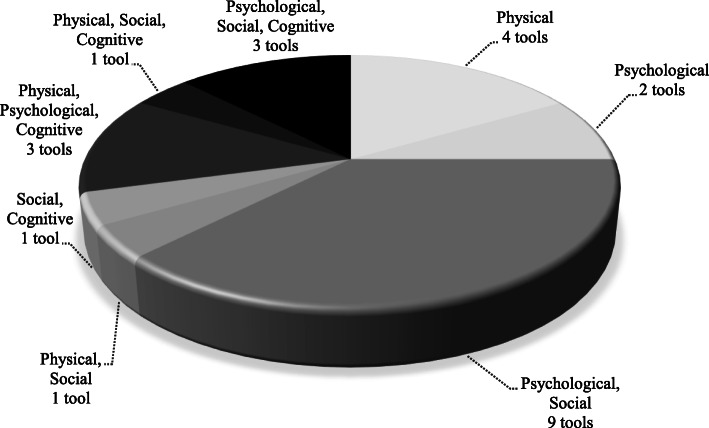
Graphical representation of the APLF domains assessed by included measures

A description of the study characteristics and their assessment instruments are presented in Table 1. The 41 studies were published between 1936 and 2020 and were conducted within the United States (*n* = 18), Netherlands (*n* = 3), South Africa (*n* = 3), Finland (*n* = 2), Italy (*n* = 2), Israel (*n* = 2), Portugal (*n* = 2), Australia (*n* = 1), Poland (*n* = 1), Canada (*n* = 1), Japan (*n* = 1), and Brazil (*n* = 1). Study location was unspecified in four studies. All relevant domains of the APLF (i.e. physical, psychological, social, and cognitive) assessed in each measure were identified (see Table 1). Tools were categorized as single domain (assessing one domain of the APLF), dual-domain (assessing two domains), and tri-domain (assessing three domains) measures. The majority of tools identified in this review assessed elements across two domains of the APLF (see Fig. 3). No single teacher proxy-report measure assessed elements in all four domains of the APLF. A detailed synthesis of how each tool (and relevant items) are aligned with individual elements of the APLF is presented in Table 4.

**Table 1:** General characteristics of studies included in the systematic review

Instrument	General construct assessed	***APLF domain(s) assessed (total no)***	Citation	Targeted age group/Grades	Sample population/ Country	Sample size (Mean age, SD)	Available translation	Completion time (minutes/seconds)	Recall period	All subscale(s) of the tool (number of items)/Total item no	Response options	Psychometric properties evaluated (statistical tests utilized)
**Single Domain Measures**												
Motor Observation Questionnaire for Teachers (MOQ-T)	Fine, gross, and perceptual motor behaviour	*Physical (1)*	Schoemaker et al. [45]	5–11 years	Children in three rehabilitation centres and the general population in Netherlands	182 children. 91 children referred for motor problems to a rehabilitation centre (Mean age 7.7, SD 1.5) and 91 comparison children (Mean age 7.6, SD 1.5). Proxy reporting, total teacher number unspecified	English	NS	NS	General Motor functioning and Handwriting/fine motor control. 18 items	Four-point scale (1 = “never true”; 4 = “always true”)	*Criterion validity* (ROC curve and Spearman correlations); *Convergent validity* (Spearman correlations); and *Discriminant validity* (ANOVA)
	Fine, gross, and perceptual motor behaviour	*Physical (1)*	Giofrè et al. [46]	5–11 years	Children in Grades 2–5 in Northern Italian schools	363 children. 102 children in Grade 2 (Mean age 92.82 months, SD 3.49), 80 in Grade 3 (Mean age 105.09 months, SD 3.76), 81 in Grade 4 (Mean age 116.58 months, SD 4.81), and 100 in Grade 5 (Mean age 128.79 months, SD 3.44). Proxy reporting 1 to 3 teachers per child; total teacher number not specified	Italian	NS	NS	General Motor functioning and Handwriting/fine motor control. 18 items	Four-point scale (1 = “never true”; 4 = “always true”)	*Structural validity* (EFA using principal axis factor method with a Promax oblique rotation and CFA); and *Internal consistency* (Cronbach’s Alpha)
	Fine, gross, and perceptual motor behaviour	*Physical (1)*	Asunta et al. [47]	6–9 years	Children in pre- and elementary schools in central Finland and five other Finnish territories	Sample 1: 193 children (Mean age 9 years 5 months, NR) Sample 2: 850 children (Mean age 7 years 7 months, NR). Proxy-reporting by 27 teachers (Sample 1)	Finnish	3.3 min	NS	General Motor functioning and Handwriting/fine motor control. 18 items	Four-point scale (1 = “never true”; 4 = “always true”)	*Structural validity* (PCA with varimax rotation and CFA using the maximum likelihood robust estimation method); *Criterion validity* (ROC curve and Spearman’s rho); *Known-groups validity* (Mann Whitney); and *Internal consistency* (Cronbach’s Alpha)
	Fine, gross, and perceptual motor behaviour	*Physical (1)*	Nowak, Schoemaker [48]	5–11 years	Children in two primary schools Wroclaw, Poland	348 children (Mean age 8.1, SD 1.9), in addition to 31 children referred from local private therapy centres (Mean age 7.8, SD 2.1). Proxy reporting by two physical education teachers	Polish	NS	NS	General Motor functioning and Handwriting/fine motor control. 18 items	Four-point scale (1 = “never true”; 4 = “always true”)	*Structural validity* (PCA with varimax rotation); *Criterion validity* (Spearman’s rank correlations and ROC curve); and *Internal consistency* (Cronbach’s Alpha)
Movement Assessment Battery for Children - 2 Checklist (MABC-2 Checklist)	Motor skills	*Physical (1)*	Schoemaker et al. [49]	5–11 years	Children in a Dutch sample in Netherlands	383 children (Mean age 6 years 9 months, NR). Proxy reporting, total teacher number unspecified. Each teacher rated five children	Dutch	NS	NS	Gross motor coordination skills (5), Ball skills (5), Recreation skills (5), fine motor skill (5), rhythmic skills (5), dynamic balance (5). 30 items (motor part)	Four-point scale (0 = “very well”; 3 = “not close”)	*Structural validity* (PCA with Varimax rotation); *Criterion validity* (Spearman rank order correlation); *Convergent validity* (Spearman rank order correlation); *Discriminant validity* (logistic regression, ROC curve, ANOVA, *t*-test); and *Internal consistency* (Cronbach’s Alpha)
	Motor skills	*Physical (1)*	Kita et al. [50]	5–11 years	Children from 16 elementary schools in a community population in Japan	3852 children. Proxy reporting: 484 valid responses following data cleaning (NR, NR). Proxy reporting, total teacher number unspecified.	Japanese	NS	NS	Gross motor coordination skills (5), Ball skills (5), Recreation skills (5), fine motor skill (5), rhythmic skills (5), dynamic balance (5). 30 items (motor part)	Four-point scale (0 = “very well”; 3 = “not true”)	*Structural validity* (confirmatory factor analysis); *Cross-cultural validity* (one-way ANOVA); and *Internal consistency* (Cronbach’s alpha)
	Motor skills	*Physical (1)*	Capistrano et al. [51]	5–11 years	School children in Florianópolis, Brazil	40 children (Mean age 8.93 years, SD 1.22 for boys and 9.04 years, SD 1.00 for girls). Proxy reporting by 16 teachers (14 classroom teachers and 2 physical education teachers)	Portuguese	NS	NS	Gross motor coordination skills (5), Ball skills (5), Recreation skills (5), fine motor skill (5), rhythmic skills (5), dynamic balance (5). 30 items (motor part)	Four-point scale (0 = “Not at all” (0); 1 = “some”; 3 = “high”)	*Criterion validity* (one-way ANOVA); *Convergent validity* (Spearman correlation)
	Motor skills	*Physical (1)*	De Milander et al. [52]	5–11 years	Children in seven mainstream schools in Free State province, South Africa	323 children (Mean age 6 years 8 months, SD 0.4). Proxy reporting by 28 teachers	English	NS	Six months	Gross motor coordination skills (5), Ball skills (5), Recreation skills (5), fine motor skill (5), rhythmic skills (5), dynamic balance (5). 30 items (motor part)	Four-point scale (0 = “very well”; 1 = “just ok”; 2 = “almost” and 3 = “not close”)	*Criterion validity* (Kappa coefficient k-)
Pictorial Scale of Perceived Water Competence (PSPWC)	Water skills	*Physical (1)*	De Pasquale et al. [53]	4–8 years	Children in four swim centres in the western part of Melbourne, Australia	51 children (Mean age 6.64 years, SD 1.49). 15 swim teachers (Mean age 27.30, SD 8.44) recruited for content validity	English	5 min for parents and 20 min for children. NS for teachers	NS	17 swimming scenarios varying in skill complexity. Each scenario is represented by three cartoon images	1–3 Likert scale (1 = “not able to do the skill”; 2 = “skill in progress”; 3 = “able to do the skill”	*Content validity*
Reiss Motivation Profile for children (Child RMP)	Intrinsic motivation	*Psychological (1)*	Weems et al. [54]	4–11 years	Children in elementary schools in the United States	333 children (NR, NR). Proxy reporting, total teacher number unspecified	English	10 min	NS	Competence (8), Social Contact (8), Character (8), Competition (8), Order (8), Physical Activity (8), Acceptance (8), Popularity (8), Anxiety (8), Curiosity (8). 80 items	Five-point scale (0 = “strongly disagree”; 1 = “disagree”; 2 = “neutral”; 3 = “agree”; 4 = “strongly agree”)	*Structural validity* (CFA); *Discriminant validity* (ANOVA); and *Internal consistency* (Cronbach’s Alpha)
Teacher’s Self-concept Evaluation Scale	Self-concept	*Psychological (1)*	Mocke et al. [55]	10–13 years	Children from a primary school in Western Cape, South Africa	114 children (Mean age 12.3, NR). Proxy reporting, total teacher number unspecified	NS	NS	NS	Personal Self-concept, School and Academic Self-concept, Physical Self-concept, Social Self-concept, General self-concept. NR	5-point scale; response options not specified	*Convergent validity* (correlation); *Internal consistency* (Cronbach’s Alpha)
Teen Risk Screen checklist (TRS)	Fundamental motor skills	*Physical (1)*	Kidd, Africa [56]	NS	Girls in one primary school in Stellenbosch region, South Africa	125 children (Mean age 12.12, SD 1.1). Proxy reporting by seven classroom teachers	English	30–40 min for a group of 20 children	NS	Posture and stability-Axial movement (7), Posture and stability Dynamic movement (5), Locomotor skills-single skills (5), Locomotor skills-combination (3), Manipulative skills-sending away (3), Manipulative skills-possession (2) and Manipulative skills-gaining possession (1). 26 items	Three-point scale (0 = “cannot perform according to guidelines”; 1 = “can perform but not according to guidelines”; 2 = “can perform skill”)	*Structural validity* (CFA); *Reliability* – 2-weeks test-retest (Pearson correlations and ICC, Kappa for one subscale); and *Internal consistency* (Cronbach alpha)
**Dual-Domain Measures**												
Brief Behaviour Rating Scale (BBRS)	Social behaviour	*Psychological, Social (2)*	Gresham et al. [57]	NS	Children in the Albuquerque, New Mexico, school district, United States	200 children (Mean age 7.2, SD 1.0). Proxy reporting, total teacher number unspecified	English	15 s per item, 3 min for entire scale	NS	Assertion (3); Cooperation (4); Self-Control (1); Hyperactivity (2), Externalizing (1); Academic Competence (1). 12 items	NS	*Convergent validity* (Pearson correlations); *Reliability – 3 months test-retest* (correlation); and *Internal consistency* (Cronbach’s alpha)
Devereux Student Strengths Assessment (DESSA)	Social and emotional competence	*Psychological, Social (2)*	Nickerson, Fishman [58]	Grades K - 8	NS	Number of children unspecified. Proxy-reporting by 94 teachers	NS	4–8 min per child	Four weeks	Optimistic Thinking (7); Self-Management (11); Goal-Directed Behaviour (10); Self-Awareness (7); Social- Awareness (9); Personal Responsibility (10); Decision Making (8); Relationship Skills (10). 72 items	Five-point scale (0 = “Occasionally”; 1 = “Never” 2 = “Rarely”; 3 = “Frequently”; 4 = “Very Frequently”)	*Convergent validity* (Pearson product moment correlations)*;* and *Divergent validity* (Pearson product moment correlations)
	Social and emotional competence	*Psychological, Social (2)*	Doromal et al. [59]	Grades K - 8	Children in an urban, Southeastern school district in the United States	313 children (Mean age 5.60 years, SD 0.30). Proxy reporting, total teacher number unspecified	English	NS	NS	Self-Awareness (7); Self-Management (11); Social Awareness (9); Decision-Making (8); Relationship Skills (10). 45 items	Five-point scale (0 = “Occasionally”; 1 = “Never” 2 = “Rarely”; 3 = “Frequently”; 4 = “Very Frequently”)	*Structural validity* (CFA); *Convergent validity* (correlations); and *Discriminant validity* (correlations)
Emotion Regulation Checklist (ERC)	Emotion regulation	*Psychological, Social (2)*	Molina et al. [60]	NS	Children in kindergarten and elementary schools in several regions in Italy	910 children (Mean age 5.77 years, SD 2.26). Proxy reporting, total teacher number unspecified	Italian	NS	NS	Emotion Regulation (8); Lability/Negativity (15). 24 items.	Four-point scale (1 = “Almost always”; 4 = “Never”)	*Structural validity* (EFA and CFA); *Internal consistency* (Cronbach’s Alpha)
Multisource Assessment of Social Competence Scale (MASCS)	Social competence	*Psychological, Social (2)*	Junttila et al. [61]	Grades K - 12	Children in 15 elementary schools in southern Finland, Finland	Cohort 1: 446 mainstream children (Mean age 10 years 5 months, SD 6.1 months) and 61 special education children (Mean age 11 years 6 months, SD 13.4 months) Cohort 2: 445 mainstream children (Mean age 10 years 2 months, SD 6.4 months) and 33 special education children (Mean age 10 years 10 months; SD 9.1 months). Proxy reporting, total teacher number unspecified	Finnish	NS	NS	Cooperating skills (5), Empathy (3), Impulsivity (3), and Disruptiveness (4). 16 items	Four-point scale (1 = “never”; 2 = “rarely”; 3 = “frequently”; 4 = “very frequently”)	*Structural validity* (CFA); *Convergent validity* (correlations); *Known-groups validity* (t-tests); and *Internal consistency* (Cronbach’s alpha)
Pictorial Scale of Perceived Competence and Social Acceptance for Young Children-Teacher (PSPCSA-T)	Perceived competence	*Physical, Social (2)*	Harter, Pike [62]	4–7 years. One version for pre- schoolers and kindergartners (4–5 years) and another for first and second graders (6–7 years)	NS	77 pre-schoolers, 28 kindergartners, and 38 first and second graders (NR, NR). Proxy reporting, total teacher number unspecified	English	NS	NS	Cognitive competence (6), Physical competence (6), and Peer acceptance (6). 18 items	Four-point scale (“really true”; “pretty true”; “only sort of true”; and “not very true”)	*Convergent validity* (correlations)
	Perceived competence	*Physical, Social (2)*	Strein, Simonson [63]	4–7 years. One version for pre- schoolers and kindergartners (4–5 years) and another for first and second graders (6–7 years)	Children in the United States	227 kindergarten students (NR, NR). Proxy reporting, total teacher number unspecified	English	NS	NS	Cognitive competence (6), Physical competence (6), and Peer acceptance (6). 18 items	Four-point scale (“really true”; “pretty true”; “only sort of true”; and “not very true”)	*Convergent validity* (Pearson’s correlations); and *Internal consistency* (Cronbach’s Alpha)
	Perceived competence	*Physical, Social (2)*	Garrison et al. [64]	4–7 years. One version for pre- schoolers and kindergartners (4–5 years) and another for first and second graders (6–7 years)	Children in New England, United States	83 children (NR, NR). Proxy reporting, total teacher number unspecified	English	NS	NS	Cognitive competence (6), Physical competence (6), and Peer acceptance (6). 18 items	NS	*Convergent validity* (correlations)
Social-Emotional Assets and Resilience Scale, Teacher rating form (SEARS-T)	Social and emotional competence	*Psychological, Social (2)*	Merrell et al. [65]	Grades K - 12	Children and adolescents in 23 Public and private schools in 10 states in the United States	1673 children and adolescents (NR, NR). Proxy-reporting by 418 teachers (average four student rating per teacher)	English	12–18 min (average 15 min)	Six months	Responsibility (10), Social competence (12), Self-regulation (13), and Empathy (6). 41 items	Four-point scale (0 = “never true”; 1 = “sometimes true”; 2 = “often true”; and 3 = “always/almost always true”)	*Structural validity* (EFA using principle axis factor with Oblimin oblique rotation, CFA using maximum likelihood estimation); *Convergent validity* (Bivariate Pearson product-moment correlations); *Known-groups validity* (independent samples t-test, one-way ANOVA); and *Internal consistency* (Cronbach’s Alpha)
	Social and emotional competence	*Psychological, Social (2)*	Romer, Merrell [66]	Grades K - 12	Children in two elementary schools in Washington, United States	118 children in Grades K - 5 (NR, NR). Proxy-reporting by 30 teachers (four student ratings per teacher)	English	NS	Six months	Responsibility (10), Social competence (12), Self-regulation (13), and Empathy (6). 41 items	Four-point scale (0 = “never true”; 1 = “sometimes true”; 2 = “often true”; and 3 = “always/almost always true”)	*Test-retest reliability* (Pearson product-moment correlations)
	Social and emotional competence	*Psychological, Social (2)*	Figueiredo et al. [67]	Grades K - 12	Children in schools in the Norther region of Portugal	235 children (116 boys and 119 girls) aged between 5 and 10 years (M = 7.51, SD = 1.63). Proxy reporting by 46 teachers.	Portuguese	NS	Six months	Responsibility (10), Social competence (12), Self-regulation (13), and Empathy (6). 41 items	Four-point scale (0 = “never true”; 1 = “sometimes true”; 2 = “often true”; and 3 = “always/almost	*Structural validity* (CFA); *Internal consistency* (Cronbach’s alpha); *Convergent validity* (Pearson’s correlation); and *Known-groups validity* (t-test)
Social Skills Improvement System Social Emotional Learning Edition Rating Forms (SSIS SEL RF) – Teacher version	Social emotional competence	*Psychological, Social (2)*	Frank Gresham et al. [68]	3–18 years	Children in the United States	200 children (NR, NR). Proxy reporting by 146 elementary teachers	English	NS	NS	Self-Awareness, Self-Management, Social Awareness, Relationship Skills, Responsible Decision-Making, and Academic Competence. 58 items.	Four-point Likert scale (0 = “Never”; 1 = “Sometime”; 2 = “Often”; and 3 = “Always”)	*Structural validity* (CFA); *test-retest reliability* (), Internal consistency; Interrater reliability
Teacher-Child Rating Scale (T-CRS)	Social and emotional competence	*Psychological, Social (2)*	Hightower et al. [69]	NS	NS	1379 children. Proxy reporting by 200 teachers Study 1 353 children from 67 K-3 classes. Each teacher rated about 6 children. Proxy reporting by 200 teachers Study 2 1026 K-6 children	NS	NS	NS	Acting out (6), Shy-anxious (6), Learning (6), Reaction to Limits/Frustration tolerance (6), Assertive social skills (6), and Good student/task orientation (6). 36 items	NS	*Structural validity* (PCA with varimax and oblique rotations); *Convergent validity* (correlations); *Known-groups validity* (one-way MANOVA); *Reliability* (test-retest); and *Internal consistency* (Cronbach’s Alpha)
Teacher Questionnaire (TQ)	Self-concept	*Psychological, Social (2)*	Jensen et al. [70]	NS	Children in third grade in a school in southern California, United States	62 children. Proxy reporting by three teachers.	English	NS	NS	Relationship with peers I (1), Relationship with peers II (1), Intellectual self-image (1), Helpfulness (2), Physiological self-image (2), Adult acceptance (2), Emotional self (2), and Tasks undertaken (1). 12 items	“Present” or “Absent”; score of two or more to be _socially_ desirable	*Convergent validity* (Phi coefficients)
Teacher Rating of Social Efficacy	Social behaviour	*Social, Cognitive (2)*	Wheeler, Ladd [71]	Grades 3–5	Children in elementary school in New York State and Indiana, United States	138 children in New York State and 107 children in Indiana. Proxy reporting by teacher, number not specified	English	NS	NS	Conflict (6), Non-conflict (5). 11 items	Four-point scale (HARD!, hard, easy, EASY!)	*Structural validity* (PCA); *Convergent validity* (Correlations); *Test-retest reliability* (correlation); and *Internal consistency* (Cronbach’s Alpha)
Winnetka Scale for Rating School Behaviour	School behaviour	*Psychological, Social (2)*	Van Alstyne [72]	Nursery to Grade 6	Children in Winnetka Public Schools, Emergency Nursery Schools in Chicago and rural schools in Kansas, United States	1200 children. Proxy reporting by their teachers, no not specified. Eight teachers for reliability	English	NS	2 months or more	Cooperation, Social Consciousness, Emotional Adjustment, Leadership, and Responsibility. 30 situations	Ratings weighted from zero to ten	*Structural validity* (Multiple factor analysis using Thurstone’s method); *Convergent validity* (correlations); and *Test-retest reliability* (Pearson’s correlations)
	School behaviour	*Psychological, Social (2)*	Leton et al. [73]	NS	NS	250 pupils in Grade 2 – Grade 6. Proxy reporting by ten teachers (nine females and one male)	English	NS	NS	13 situations: Taking turns; Cooperation on group projects; Sacrifice for group; Application to social tasks; Emotional tone; Independence of adult approval; Reaction to failure; Self-confidence; Direction of group tasks; Group leadership; Continuing with academic tasks; Self-reliance; Organization of work	Ratings weighted from zero to ten	*Structural validity* (PCA with varimax rotation); *Known-groups validity* (mean differences)
**Tri-Domain Measures**												
Children Activity Scales for Teachers (CHAS-T)	Motor behaviour	*Physical, Psychological, Cognitive (3)*	Rosenblum [74]	4–8 years	Israeli children	Sample 1: 355 children. Proxy reporting by 14 teachers Sample 2: Two groups of 30 children each (TD and DCD)	Originally in Hebrew but translated to English/ Israel	5–10 min per child	NS	Gross Motor Skills (6), Fine Motor Skills (5), and Organization in Space and Time (10). 21 items	Five- point scale (5 = “very well”; 1 = “less adequately”)	*Content and face validity; Structural validity* (PCA with varimax rotation); *Criterion validity* (correlations); *Convergent validity* (correlations); *Discriminant validity* (discriminant analyses); and *Internal consistency* (Cronbach’s alpha)
Gross Motor Rating Scale (GMRS)	Gross motor ability	*Physical, Psychological, Cognitive (3)*	Netelenbos [75]	NS	Children in schools in a suburban region of a Dutch city in Netherlands	Study 1: 132 children (Mean age 5.3 years, SD 15.1 months). Proxy-reporting by their teachers, number not specified. Study 2: 94 children (Mean age 5.2 years, SD 12.5 months) Study 3: 43 children (Mean age 6.7 years, SD 7.7 months)	English	NS	NS	Gross Motor Skills (10); Physical qualities (7); Movement motivation (3). 20 items	Five-point scale (1 = “poor”; 2 = “moderate”; 3 = “adequate”; 4 = “good” and 5 = “excellent”)	*Structural validity* (PCA); *Criterion validity* (Pearson correlation); *Convergent validity* (correlations); *Reliability* (interrater [correlations], *test-retest* [correlations]); *Internal Consistency* (Cronbach’s alpha)
Harter’s Teacher’s Rating Scale of Child’s Actual Behaviour (TRS)	Perceived competence	*Physical, Social, Cognitive (3)*	Cole et al. [76]	NS	Children in one of nine public schools in a midsize, midwestern school district in United States	897 children (Mean age 8.9, SD 0.5 for third graders and Mean age 11.9, SD 0.5 for sixth graders). Proxy-reporting by 49 teachers	English	NS	NS	Scholastic Competence (3), Social Acceptance (3), Athletic Competence (3), Physical Appearance (3), and Behavioural Conduct (3). 15 items.	Four-point scale. Two-step process. Teacher selects one of two statements that describes the child. Teacher indicates whether their choice is “Sort of true” or “Really true” about the child	*Convergent validity* (Inter-battery factor analysis)
	Perceived competence	*Physical, Social, Cognitive (3)*	Cole et al. [77]	NS	Children in nine public schools in a mid-size, midwestern school district in United States	724 children (NR, NR). Proxy reporting by 49 teachers	English	NS	NS	Scholastic Competence (3), Social Acceptance (3), Athletic Competence (3), Physical Appearance (3), and Behavioural Conduct (3). 15 items.	Four-point scale. Two-step process. Teacher selects one of two statements that describes the child. Teacher indicates whether their choice is “Sort of true” or “Really true” about the child	*Structural validity* (CFA); *Convergent validity* and *Discriminant validity* (Multigroup-Multitrait- multimethod confirmatory factor analysis)
	Perceived competence	*Physical, Social, Cognitive (3)*	Cole et al. [78]	NS	Children in nine elementary schools and two middle schools in the United States	1228 children and adolescents (Mean age 8.9 years, SD 0.5 for third graders and 11.9 years, SD 0.5 for sixth graders). Proxy reporting, total teacher number unspecified	English	NS	NS	Scholastic Competence (3), Social Acceptance (3), Athletic Competence (3), Physical Appearance (3), and Behavioural Conduct (3). 15 items.	Four-point scale. Two-step process. Teacher selects one of two statements that describes the child. Teacher indicates whether their choice is “Sort of true” or “Really true” about the child	*Convergent validity* and *Discriminant validity* (Multitrait-multimethod analysis)
Health Resources Inventory (HRI)	Personal and social competence	*Psychological, Social, Cognitive (3)*	Gesten [79]	Primary grade children	Children in 12 schools (five in the Rochester City School District and seven in two adjacent county districts) in United States	592 children (NR, NR). Proxy-reporting by 65 teachers	English	NS	NS	Good student (10), Gutsy (7), Peer Sociability (10), Rules (7), and Frustration Tolerance (20). 54 items	Five-point scale (1 = “Not at all”; 5 = “Very well”)	*Structural validity* (PCA with varimax and oblique rotation); *Convergent validity* (correlations); *Discriminant validity* (t-test); *Known-groups validity* (ANOVA); *Test-retest reliability* (reliability coefficients)
Social and Emotional Competencies Evaluation Questionnaire Teacher’s version – Short Form (QACSE-P-SF)	Social and emotional competencies	*Psychological, Social, Cognitive (3)*	Coelho et al. [80]	Grades 4–9	Children in five public schools in the Lisbon District of Portugal	657 children (Mean age 11.3 years, SD 1.8). Proxy reporting by 39 teachers	Portuguese	Five minutes per child	NS	Social Awareness (5), Self-control (5), Social Isolation (5), Social Anxiety (5), Responsible Decision Making (5), Relationship Skills (5). 30 items	Four-point scale (A = “never”; B = “sometimes”; C = “frequently”; D = “always”)	*Structural validity* (PCA with Varimax rotation - Kaiser normalization; CFA using Maximum Likelihood estimation); *Known-groups validity* (t-tests, one-way ANOVA); *Test-retest reliability* (correlations); and *Internal consistency* (Cronbach’s alpha)
Social Skills Rating Scale (SSRS-T)	Social skills	*Psychological, Social, Cognitive (3)*	Clark et al. [81]	NS	Children in two schools in a metropolitan district in the United States	194 children (Mean age 9 years 5 months, NR). Proxy-reporting by 26 teachers (five girls and five boys rating per teacher)	English	NS	NS	Social initiation (15), Academic performance (13), Cooperation (17), and Peer reinforcement (7). 52 items (frequency dimension)	Five-point scale (1 = “never true”; 5 = “frequently”)	*Structural validity* (PCA); *Convergent validity* (Correlations); and *Internal consistency* (Cronbach’s alpha)
	Social skills	*Psychological, Social, Cognitive (3)*	Frank M Gresham et al. [82]	NS	Children in in a school district in south eastern Louisiana, United States	250 children (NR, NR). Proxy reporting by 43 black and 82 white regular classroom teachers	English	NS	NS	50 items (frequency dimension)	Response option for frequency (2 = “often true”; 1 = “sometimes true”; 0 = “never true”)	*Structural validity* (PCA); *Known-groups validity* (); and *Internal consistency* (Cronbach’s alpha)
	Social skills	*Psychological, Social, Cognitive (3)*	Elliott et al. [83]	NS	Children in elementary schools in Louisiana, United States	60 children (NR, NR). Proxy reporting by six teachers	English	NS	NS	50 items. Two dimensions (frequency and importance) of social behaviour	Response option for frequency dimension same as above. Importance (2 = “critical for success in my classroom”; 1 = “important for success in my classroom”; and 0 = “unimportant for success in my classroom”)	*Divergent validity* (Correlations); *Known-groups validity* (MANOVA); *Reliability* (*test-retest* [reliability coefficients], *interrater* [correlations]); and *Internal Consistency* (Cronbach’s alpha)
Teacher Estimation of Activity Form (TEAF)	Children’s motor ability, participation and self-efficacy towards physical activity	*Physical, Psychological, Cognitive (3)*	Faught et al. [17]	NS	Children in 15 schools from the District School Board of Niagara in Ontario, Canada	502 children (NR, NR). Proxy reporting, total teacher number unspecified	English	10 min per child	NS	Gross motor ability. 10 items	Five-point scale (1 = “well below average”; 2 = “somewhat below average”; 3 = “average”; 4= “somewhat above average”; and 5 = “well above average”)	*Structural validity* (factor analysis); *Criterion validity* (ROC curve); *Convergent validity* (correlations); and *Internal consistency* (Cronbach’s alpha)
	Children’s motor ability, participation and self-efficacy towards physical activity	*Physical, Psychological, Cognitive (3)*	Sara Rosenblum, Engel-Yeger [84]	NS	Children in mainstream public schools in Northern Israel	123 children, 68 TD and 55 DCD (NR, NR). Proxy- reporting by 6 physical education teachers	Hebrew	NS	NS	Gross motor ability. 10 items	Five-point scale (1 = “well below average”; 2 = “somewhat below average”; 3 = “average”; 4 = “somewhat above average”; and 5 = “well above average”)	*Structural validity* (factor analysis); *Criterion validity* (Pearson’s correlations); *Known-groups validity* (t-test, MANOVA, discriminant analysis); and *Internal consistency* (Cronbach’s Alpha)

*ANOVA* analysis of variance, *CFA* confirmatory factor analysis, *DCD* developmental coordination disorder, *EFA* exploratory factor analysis, *ICC* Intraclass correlation coefficient, *MANOVA* multivariate analysis of variance, *NR* not reported, *NS* not specified, *PCA* principal component analysis, *ROC* receiver operator characteristic, *SD* standard deviation, *TD* typically developing

For “single domain measures”, four tools assessed elements exclusively in the physical domain: the Motor Observation Questionnaire for Teachers (MOQ-T) [45–48]; Movement Assessment Battery for Children-2 Checklist (MABC-2 Checklist) [49–52]; Pictorial Scale of Perceived Water Competence (PSPWC) [53]; and Teen Risk Screen checklist (TRS) [54]. Another two tools were related only to the psychological domain: Reiss Motivation Profile for children (Child RMP) [55]; and Teacher’s Self-concept Evaluation Scale [56].

“Dual-domain measures” included the Brief Behaviour Rating Scale (BBRS) [57]; Devereux Student Strengths Assessment (DESSA) [58, 59]; Emotion Regulation Checklist (ERC) [60]; Multisource Assessment of Social Competence Scale (MASCS) [61]; Pictorial Scale of Perceived Competence and Social Acceptance for Young Children-Teacher (PSPCSA-T) [62–64]; Social-Emotional Assets and Resilience Scale, Teacher rating form (SEARS-T) [65–67]; Social Skills Improvement System Social Emotional Learning Edition Rating Forms (SSIS SEL RF) – Teacher version [68]; Teacher-Child Rating Scale (T-CRS) [69]; Teacher Questionnaire (TQ) [70]; Teacher Rating of Social Efficacy [71]; and Winnetka Scale for Rating School Behaviour [72, 73] (See Fig. 3 and Table 1).

Tools that straddled across three domains “tri-domain measures” of the framework included the Children Activity Scales for Teachers (CHAS-T) [74]; Gross Motor Rating Scale (GMRS) [75]; Harter’s Teacher’s Rating Scale of Child’s Actual Behaviour (Harter’s TRS) [76–78]; Health Resources Inventory (HRI) [79]; Social and Emotional Competencies Evaluation Questionnaire Teacher’s version (Short Form) (QACSE-P-SF) [80]; Social Skills Rating Scale (SSRS-T) [81–83]; and Teacher Estimation of Activity Form (TEAF) [17, 84] (See Fig. 3 and Table 1).

Furthermore, there was a considerable degree of homogeneity in relation to the targeted age group/grades for identified tools. Most tools spanned the entire age range (i.e. for children between 5 and 12 years) and thus were suitable for both younger and older children. Tool completion times were not often reported but when reported, completion times ranged between three and 15 min per child. Scales ranged from 10 [17, 84] to 80 items [54]. The 41 studies assessed a median of 3 out of the nine measurement properties recognized by the COSMIN. The most commonly reported psychometric properties were construct validity (*n* = 32; 78% of studies), structural validity (*n* = 26; 63% of studies), and internal consistency (*n* = 25; 61% of studies). Statistical tests utilized to evaluate measurement properties varied across the review. For instance, confirmatory factor analysis was the most frequently used statistical approach for studies reporting on structural validity whereas correlations were used for hypothesis testing for construct validity. Construct validity was mostly tested by comparing scores obtained for a tool with another measure assessing a similar construct. On the other hand, criterion validity was evaluated by comparing scores obtained for a tool with a gold standard measure. Tool development studies were conducted for eight measures including the BBRS [57], CHAS-T [74], GMRS [75], HRI [79], SEARS-T [65], SSRS-T [81], T-CRS [69], and Winnetka Scale for Rating School Behaviour [72]. Content validity was only reported for two tools (CHAS-T and PSPWC) [53, 74].

### Psychometric properties

#### Methodological quality assessment

Table 2 details the methodological quality assessment of the 41 studies included in the review.

**Table 2:** Summary of methodological quality assessment for included studies

Instrument name (Citation)	Tool Development	Measurement property methodological quality per study								
		Content validity	Structural validity	Internal consistency	Cross-cultural validity	Reliability	Measurement error	Criterion validity	Hypothesis testing	Responsiveness
**Single domain Measures**										
Motor Observation Questionnaire for Teachers (MOQ-T) [45]	NR	NR	NR	NR	NR	NR	NR	V	V	NR
Motor Observation Questionnaire for Teachers (MOQ-T) [46]	NR	NR	I	I	NR	NR	NR	NR	NR	NR
Motor Observation Questionnaire for Teachers (MOQ-T) [47]	NR	NR	I	V	NR	NR	NR	V	D	NR
Motor Observation Questionnaire for Teachers (MOQ-T) [48]	NR	NR	D	V	NR	NR	NR	V	NR	NR
Movement Assessment Battery for Children - 2 Checklist (MABC-2 Checklist) [49]	NR	NR	A	I	NR	NR	NR	V	V	NR
Movement Assessment Battery for Children - 2 Checklist (MABC-2 Checklist) [50]	NR	NR	V	V	D	NR	NR	NR	NR	NR
Movement Assessment Battery for Children - 2 Checklist (MABC-2 Checklist) [51]	NR	NR	NR	NR	NR	NR	NR	I	A	NR
Movement Assessment Battery for Children - 2 Checklist (MABC-2 Checklist) [52]	NR	NR	NR	NR	NR	NR	NR	I	NR	NR
Pictorial Scale of Perceived Water Competence (PSPWC) [53]	NR	D	NR	NR	NR	NR	NR	NR	NR	NR
Reiss Motivation Profile for children (Child RMP) [54]	NR	NR	D	V	NR	NR	NR	NR	A	NR
Teacher’s Self-concept Evaluation Scale [55]	NR	NR	NR	I	NR	NR	NR	NR	V	NR
Teen Risk Screen checklist (TRS) [56]	NR	NR	D	V	NR	A	NR	NR	NR	NR
**Dual-Domain Measures**										
Brief Behaviour Rating Scale (BBRS) [57]	I	NR	NR	I	NR	I	NR	NR	V	NR
Devereux Student Strengths Assessment (DESSA) [58]	NR	NR	NR	NR	NR	NR	NR	NR	V	NR
Devereux Student Strengths Assessment (DESSA) [59]	NR	NR	D	NR	NR	NR	NR	NR	V	NR
Emotion Regulation Checklist (ERC) [60]	NR	NR	D	V	NR	NR	NR	NR	NR	NR
Multisource Assessment of Social Competence Scale (MASCS) [61]	NR	NR	V	V	NR	NR	NR	NR	A	NR
Pictorial Scale of Perceived Competence and Social Acceptance for Young Children-Teacher (PSPCSA-T) [62]	NR	NR	NR	NR	NR	NR	NR	NR	A	NR
Pictorial Scale of Perceived Competence and Social Acceptance for Young Children-Teacher (PSPCSA-T) [63]	NR	NR	NR	V	NR	NR	NR	NR	V	NR
Pictorial Scale of Perceived Competence and Social Acceptance for Young Children-Teacher (PSPCSA-T) [64]	NR	NR	NR	NR	NR	NR	NR	NR	V	NR
Social-Emotional Assets and Resilience Scale, Teacher rating form (SEARS-T) [65]	D	NR	V	V	NR	NR	NR	NR	A	NR
Social-Emotional Assets and Resilience Scale, Teacher rating form (SEARS-T) [66]	NR	NR	NR	NR	NR	A	NR	NR	NR	NR
Social-Emotional Assets and Resilience Scale, Teacher rating form (SEARS-T) [67]	NR	NR	D	V	NR	NR	NR	NR	V	NR
Social Skills Improvement System Social Emotional Learning Edition Rating Forms (SSIS SEL RF) – Teacher version [68]	NR	NR	A	V	NR	D	NR	NR	NR	NR
Teacher-Child Rating Scale (T-CRS) [69]	I	NR	A	V	NR	D	NR	NR	A	NR
Teacher Questionnaire (TQ) [70]	NR	NR	NR	NR	NR	NR	NR	NR	I	NR
Teacher Rating of Social Efficacy [71]	NR	NR	D	I	NR	D	NR	NR	V	NR
Winnetka Scale for Rating School Behaviour [72]	I	NR	D	NR	NR	I	NR	NR	D	NR
Winnetka Scale for Rating School Behaviour [73]	NR	NR	A	NR	NR	NR	NR	NR	A	NR
**Tri-domain Measures**										
Children Activity Scales for Teachers (CHAS-T) [74]	I	D	A	I	NR	NR	NR	V	V	NR
Gross Motor Rating Scale (GMRS) [75]	I	NR	D	I	NR	D	NR	V	I	NR
Harter’s Teacher’s Rating Scale of Child’s Actual Behaviour (TRS) [76]	NR	NR	NR	NR	NR	NR	NR	NR	A	NR
Harter’s Teacher’s Rating Scale of Child’s Actual Behaviour (TRS) [77]	NR	NR	V	NR	NR	NR	NR	NR	A	NR
Harter’s Teacher’s Rating Scale of Child’s Actual Behaviour (TRS) [78]	NR	NR	NR	NR	NR	NR	NR	NR	V	NR
Health Resources Inventory (HRI) [79]	D	NR	A	NR	NR	D	NR	NR	A	NR
Social and Emotional Competencies Evaluation Questionnaire Teacher’s version – Short Form (QACSE-P-SF) [80]	NR	NR	V	V	NR	I	NR	NR	V	NR
Social Skills Rating Scale (SSRS-T) [81]	I	NR	I	I	NR	NR	NR	NR	A	NR
Social Skills Rating Scale (SSRS-T) [82]	NR	NR	A	V	NR	NR	NR	NR	V	NR
Social Skills Rating Scale (SSRS-T) [83]	NR	NR	NR	V	NR	D	NR	NR	V	NR
Teacher Estimation of Activity Form (TEAF) [17]	NR	NR	A	V	NR	NR	NR	V	V	NR
Teacher Estimation of Activity Form (TEAF) [84]	NR	NR	D	V	NR	NR	NR	V	V	NR

*NR* Not Reported, *V* Very Good, *A* Adequate, *D* Doubtful, *I* Inadequate

#### Single domain measures

The MOQ-T and MABC-2 Checklist were each evaluated in four studies [45–52]; while one study each assessed the Child RMP [54], PSPWC [53], Teacher’s Self-Concept Evaluation Scale [55], and TRS [56]. No measure assessing a single domain of the APLF reported on *tool development*, *responsiveness*, and *measurement error*. Content validity assessed for the PSPWC [53] obtained an Doubtful rating [53]. *Structural validity* ratings were generally low with studies rated as Inadequate (*n* = 2) [46, 47] or Doubtful (*n* = 3) [48, 54, 56]. Only two studies were rated as Adequate [49] and Very Good [50]. *Cross-cultural validity*, assessed in one study, received a Doubtful rating [50]. Contrariwise, studies assessing *criterion validity* mostly received Very Good (*n* = 4) ratings [45, 47–49], with only two studies being rated as Inadequate [51, 52]. For *construct validity*, most studies received favourable ratings of Very Good (*n* = 3) [45, 49, 55] or Adequate (*n* = 2) [51, 54], and only one study was rated as Doubtful [47]. Regarding measurement properties relating to reliability, one study examined the *test-retest* of the TRS and was rated as Adequate [56]. *Internal consistency* had mixed ratings; five studies were rated as Very Good [47, 48, 50, 54, 56], while three were Inadequate [46, 49, 55]. Overall, four single-domain tools (i.e. MOQ-T, MABC-2 Checklist, Child RMP, TRS) obtained consistent ratings of “Very Good” or “Adequate” for methodological quality across its measurement studies.

#### Dual-domain measures

Seventeen studies evaluated dual-domain measures [57–73]. For these measures, most measurement properties (*content validity*, *cross-cultural validity*, *measurement error*, *criterion validity*, *responsiveness)* were unreported. All studies providing information on *tool development* received ratings of either Inadequate (*n* = 3) [57, 69, 72] or Doubtful (*n* = 1) [65]. Conversely, *construct validity* was rated as Very Good (*n* = 7) [57–59, 63, 64, 67, 71] or Adequate (*n* = 5) [61, 62, 65, 69, 73]; only two studies were rated as Doubtful [72] and Inadequate [70]. Studies on *structural validity* received mixed ratings of Very Good (*n* = 2) [61, 65], Adequate (*n* = 3) [68, 69, 73], and Doubtful (*n* = 5) [59, 60, 67, 71, 72]. Furthermore, the majority of studies on *internal consistency* rated highly as Very Good (*n* = 7) [60, 61, 63, 65, 67–69]; while only two were Inadequate [57, 71]. *Reliability* studies were rated as Adequate (*n* = 1) [66], Doubtful (*n* = 3) [68, 69, 71], and Inadequate (*n* = 2) [57, 72]. Overall, six dual-domain tools (i.e. DESSA, MASCS, PSPCSA-T, SEARS-T, SSIS SEL RF Teacher, T-CRS) obtained consistent ratings of “Very Good” or “Adequate” for methodological quality across its measurement studies.

#### Tri-domain measures

Twelve studies examined tri-domain measures [17, 74–84]. Measurement properties not evaluated for any of these measures were *cross-cultural validity*, *measurement error*, and *responsiveness*. *Tool development* studies received low ratings of Inadequate (*n* = 3) [74, 75, 81] or Doubtful (*n* = 1) [79]. *Content validity* assessed in a single study for the CHAS-T was rated as Doubtful [74]. For the most part, studies on *structural validity* received high ratings of Very Good (*n* = 2) [77, 80] and Adequate (*n* = 4) [17, 74, 79, 82]. However, three studies were rated as Doubtful (*n* = 2) [75, 84] and Inadequate (*n* = 1) [81]. Similarly, majority of studies on *criterion validity* and *construct validity* were rated highly. For *criterion validity*, studies were all rated as Very Good (*n* = 4) [17, 74, 75, 84]; whereas *construct validity* studies were rated as Very Good (*n* = 7) [17, 74, 78, 80, 82–84] and Adequate (*n* = 4) [76, 77, 79, 81], with only one study rated as Inadequate [75]. *Internal consistency* studies were rated as either Very Good (*n* = 5) [17, 80, 82–84] or Inadequate (*n* = 3) [74, 75, 81]; while *reliability* studies rated lower as either Doubtful (*n* = 3) [75, 79, 83] or Inadequate (*n* = 1) [80]. Overall, four tri-domain tools (i.e. Harter’s TRS, QACSE-P-SF, SSRS-T, TEAF) obtained consistent ratings of “Very Good” or “Adequate” for methodological quality across its measurement studies.

#### Measurement property assessment of instruments

In this section, the overall rating of each tool was appraised, and Table 3 was formed. A combined synthesis of the quality of results is presented for the measures included in this review. The measurement property *structural validity* was found to be sufficient for a number of instruments including the DESSA, ERC, Harter’s TRS, MASCS, MOQ-T, and QACSPE-P-SF, where in line with the COSMIN criteria, most (i.e. 75%) single studies assessing these instruments had acceptable Root Mean Square Error of Approximation (RMSEA) (< 0.06) or comparative fit index (CFI) (> 0.95) or Standardized Root Mean Residuals (SRMR) (< 0.08) values. Inconsistent ratings were noted for the SEARS-T and MABC-2 checklist. Tools found to have insufficient structural validity were the Child RMP, SSIS SEL RF Teacher, and TRS checklist. However, the majority of tools (including the CHAS-T, GMRS, HRI, SSRS-T, TCRS, Teacher’s Rating of Social Efficacy, TEAF, and Winnetka Scale for Rating School Behaviour) were indeterminate in structural validity whereby single studies evaluating these tools utilized statistical methods such as exploratory factor analysis.

**Table 3:** Evaluating results for measurement properties against COSMIN’s updated criteria for good measurement properties

Instrument name	Citation	Structural validity (rating)	Criterion validity (rating)	Cross-cultural validity (rating)	Construct validity (rating)	Internal consistency (rating)	Reliability (rating)	Overall rating
**Single Domain Measures**								
Motor Observation Questionnaire for Teachers (MOQ-T)	Schoemaker et al. [45]	–	With Movement Assessment Battery for Children test *r* = 0.57, *p* < 0.001; AUC = 0.77, CI: 0.71–0.84; Sensitivity = 80.5%; Specificity = 62% for cut-off score > 35 (+)	–	*Convergent* With Developmental Coordination Disorder-Questionnaire *r* = −0.64, *p* < 0.001 (1+) *Discriminant* Children in referred group (49.0, SD = 11.0) versus comparison group (30.2, SD = 11.2), *F*(1,182) = 130.442, *p* < 0.001 (1?)	–	–	Structural validity + Criterion validity + Construct validity ? Internal consistency +
	Giofrè et al. [46]	EFA: 2 factors accounting for 58.26% of total variance CFA: χ^2^ (134) = 269.01, RMSEA = 0.05, SRMR = 0.05, CFI = 0.99, NNFI = 0.99, AIC = 343.01 (+)	–	–	–	Cronbach’s α 0.95 (?)	–	
	Asunta et al. [47]	PCA (varimax): 2 factors accounting for 70.5% of the total variance CFA: χ^2^ (132) = 530.90, RMSEA = 0.060, SRMR = 0.038, CFI = 0.951, TLI = 0.943, ABIC = 21,850.503 (+)	With Movement Assessment Battery for Children-2 test AUC = 0.73, 95% CI: 0.64–0.82; Sensitivity = 82.0%; Specificity = 44.4% for a cut-off score of 36 (+)	–	*Known-groups* Gender: Boys (Median = 25) and Girls (Median = 21), Mann Whitney *U* = 112,513, *z* = 6.31, *p* < 0.001, *r* = 0.216 Age: Differences between 6- and 7-year-olds (*z* = 94.70, *p* = 0.002, *r* = 0.277) and between 7- and 9-year-olds (*z* = 97.53, *p* < 0.001, *r* = 0.243), Kruskal-Wallis *H*(3) = 19.754, *p* < 0.001 (2?)	Cronbach’s α 0.96 (total), Cronbach’s α 0.96 (Motor functioning), Cronbach’s α 0.90 (Handwriting/fine motor control) (+)	–	
	Nowak, Schoemaker [48]	EFA: 3 factors accounting for 77.9% of total variance (?)	With Körperkoordinationstest für Kinder test AUC = 0.96, CI: 0.90–1.00; Sensitivity = 80%, Specificity = 94% for cut-off score ≥ 44.5; *r* = −0.789, *p* < 0.001 for control group and *r* = − 0.691, *p* < 0.001 for clinic-referred group (+)	–	–	Cronbach’s α 0.962 (total), Cronbach’s α 0.969 (Gross motor skills), Cronbach’s α 0.857 (Fine motor skills), Cronbach’s α 0.746 (General motor coordination) (+)	–	
Movement Assessment Battery for Children - 2 Checklist (MABC-2 Checklist)	Schoemaker et al. [49]	EFA: 6 factors accounting for 69% of total variance (?)	With Movement Assessment Battery for Children-2 test *r*_s_ = − 0.38, *p* < 0.001 (−)	–	*Convergent* With Developmental Coordination Disorder-Questionnaire *r*_s_ = − 0.36; *p* < 0.001 (−) *Discriminant* Checklist scores predicted motor impairment/ non-motor impairment (*B* [standard error, SE] = − 0.082 [0.015]; *p* < 0.001 [odds ratio 0.92; CI: 0.90–0.95) (?)	Cronbach’s α 0.94 (?)	–	Structural validity ± Criterion validity ? Cross-cultural validity ? Construct validity − Internal consistency ±
	Kita et al. [50]	CFA: χ^2^ /*df* = 2.355; GFI = 0.941; AGFI = 0.870, CFI = 0.987; RMSEA = 0.049 (+)	–	Significant differences between total score on original, parent rating, and teacher rating. Significant main effects in all age groups (6 year: *F*(2,92.65) = 42.75, *p* < 0.001; 7 year: *F*(2,137.56) = 45.32, *p* < 0.001; 8 year: *F*(2,140.49) = 16.43, *p* < 0.001) (?)	–	Cronbach’s α 0.973 (sections A), Cronbach’s α 0.973 (sections B) (+)	–	
	Capistrano et al. [51]	–	Significant difference between scores on the Movement Assessment Battery for Children-2 test, Checklist and the Developmental Coordination Disorder-Questionnaire (*F*_4,3_ = 810.1; *p* = 0.001) (?)	–	*Convergent* Correlation between the Checklist (classroom teacher evaluation) and the Developmental Coordination Disorder-Questionnaire *r* = − 0.28; *p* = 0.11; and between the Checklist (physical education teacher evaluation) and the Developmental Coordination Disorder-Questionnaire *r* = 0.16; *p* = 0.53 (2−)	–	–	
	De Milander et al. [52]		With Movement Assessment Battery for Children-2 test Kappa coefficient = 0.161; effect size = 0.228 (?)					
Pictorial Scale of Perceived Water Competence (PSPWC)	De Pasquale et al. [53]	–	–	–	–	–	–	Content validity *Relevance* ± *Comprehensiveness* + *Comprehensibility* +
Reiss Motivation Profile for children (Child RMP)	Weems et al. [54]	CFA: RMSEA = 0.064, 90% CI = 0.063–0.065, CFI = 0.74 (−)	–	–	*Discriminant* At-risk youth had significantly lower scores on subscales Competence, Order, Character, Social Contact, and Curiosity, but significantly higher scores on Competition and Acceptance (*ps* < 0.05) (?)	Cronbach’s α 0.90 (Popularity); Cronbach’s α 0.83 (Competence); Cronbach’s α 0.85 (Competition); Cronbach’s α 0.81 (Order); Cronbach’s α 0.86 (Anxiety); Cronbach’s α 0.92 (Character); Cronbach’s α 0.86 (Social Contact); Cronbach’s α 0.92 (Curiosity); Cronbach’s α 0.83 (Acceptance); Cronbach’s α 0.93 (Physical Activity) (?)	–	Structural validity − Construct validity ? Internal consistency ?
Teacher’s Self-concept Evaluation Scale	Mocke et al. [55]	–	–	–	*Convergent* With Preliminary Self-Concept Questionnaire *r* = 0.58, *p* < 0.01 With Self-description Questionnaire subscales *r* = 0.33 to 0.54, *p* = 0.01 (2+)	Cronbach α 0.89 (?)	–	Construct validity + Internal consistency ?
Teen Risk Screen checklist (TRS)	Kidd, Africa [56]	CFA: Posture and Stability-Axial movement: RMSEA 0.10 (*p* = 0.03), AGFI 1.00, CR 0.98, VE 0.85; Posture and stability Dynamic movement: RMSEA 0.12 (*p* = 0.05), AGFI 0.99, CR 0.95, VE 0.80; Locomotor skills-single skills: RMSEA 0.10 (*p* = 0.13), AGFI 1.00, CR 0.97, VE 0.86; Locomotor skills-combination, Manipulative skills-sending away, Manipulative skills-possession: RMSEA 0.008 (*p* = 0.74), AGFI 0.99, CR 0.79 for Locomotor skills-combination, 0.85 for Manipulative skills-sending away and 0.76 for Manipulative skills-possession, VE 0.57 for Locomotor skills-combination, 0.65 for Manipulative skills-sending away, and 0.76 for Manipulative skills-possession (−)	–	–	–	Test 1: Cronbach’s α ranged between 0.67 to 0.93 Test 2: Cronbach’s α ranged between 0.45 to 0.90 (?)	*Test-retest* ICC values: Posture and Stability-Axial movement 0.51 (0.32, 0.65); Posture and stability Dynamic movement 0.63 (0.46, 0.75); Locomotor skills-single skills 0.86 (0.76, 0.91); Locomotor skills-combination 0.74 (0.65, 0.82); Manipulative skills-sending away 0.34 (0.13, 0.51); Manipulative skills-possession 0.56 (0.42, 0.67), Manipulative skills-gaining possession (Kappa 0.36 (0.21, 0.53) (−)	Structural validity − Reliability − Internal consistency ?
**Dual Domain Measures**								
Brief Behaviour Rating Scale (BBRS)	Frank M Gresham et al. [57]	–	–	–	*Convergent* Correlation with the Teacher Report Form Total Problems (*r* = 0.51); Social Skills Rating Scale-Problem Behaviour (*r* = 0.54); Social Skills Rating System-Social Skills (*r* = − 0.59) and Social Skills Rating System-Academic Competence scale (*r* = − 0.21) (2+, 2−)	Cronbach’s α 0.70 (?)	*Test-retest* *r* = 0.71 (?)	Construct validity ± Reliability ? Internal consistency ?
Devereux Student Strengths Assessment (DESSA)	Nickerson, Fishman [58]	–	–	–	*Convergent* Correlations with Behavioural and Emotional Rating Scales–2 subscales ranged from 0.49 to 0.78, *p* < 0.01; and with the Behaviour Assessment System for Children–2 Adaptive Skills Composite were *r* = 0.92, *p* < 0.01 (2+) *Divergent* Correlations with Behaviour Assessment System for Children–2 clinical subscales with *r* = − 0.60, *r* = − 0.26, and *r* = − 0.62 for Externalizing, Internalizing, and School Problems subscales respectively (1+, 2−)	–	–	Structural validity + (five factor model) Construct validity ±
	Doromal et al. [59]	5 factors. CFI = 0.943; TLI = 0.940; RMSEA = 0.046, 90% CI = 0.042, 0.050; WRMR = 1.317 (+)	–	–	*Convergent* Correlations with Student-Teacher Relationship Scale conflict scores (*r* = − 0.61, *p* < 0.01) and closeness scores (*r* = 0.63, *p* < 0.01); Child Behaviour Rating Scale (*r* = 0.55, *p* < 0.01), and SSIS problem behaviours (*r* = − 0.52, *p* < 0.01); moderately associated with perspective taking scores (*r* = 0.22, *p* < 0.01); and weakly associated with behavioural self- regulation scores (*r* = 0.16, *p* < 0.01) (4+, 2−) *Discriminant* Social awareness scores of the DESSA were not associated with the Head-Toes- Knees-Shoulders scores (*r* = 0.07, *p* = 0.21) (?)	–	–	
Emotion Regulation Checklist (ERC)	Molina et al. [60]	CFA: χ^2^ = 845.69, *p* < 0.001, CFI = 0.98, RMSEA = 0.072, SRMR = 0.10 (+)	–	–	–	Cronbach’s α 0.79 (Emotion Regulation); Cronbach’s α 0.90 (Lability/Negativity) (+)	–	Structural validity + Internal consistency +
Multisource Assessment of Social Competence Scale (MASCS)	Junttila et al. [61]	CFA: χ^2^ (81) = 349.07; NNFI = 0.93; RMSEA = 0.08; 90% CI = 0.08, 0.09; SRMR = 0.061 (+)	–	–	*Convergent* Significant correlations with multiple sources of rating (1+, 2−) *Known-groups* Mainstream and special education: Cooperating skills (*t* = 2.71; *p* = 0.027); Empathy (*t* = 3.91; *p* < 0.001); Impulsivity (*t* = − 5.22; *p* < 0.001) and; Disruptiveness (t = − 4.35; *p* < 0.001) Gender: Cooperating skills (*t* = 7.01; *p* < 0.001); Empathy (*t* = 9.69; *p* < 0.001); Impulsivity (*t* = − 10.73; *p* < 0.001) and; Disruptiveness (*t* = − 12.88; *p* < 0.001) (2?)	Cronbach’s α 0.89 (Cooperating skills); Cronbach’s α 0.84 (Empathy); Cronbach’s α 0.88 (Impulsivity); Cronbach’s α 0.89 (Disruptiveness) (+)	–	Structural validity + Construct validity ± Internal consistency +
Pictorial Scale of Perceived Competence and Social Acceptance for Young Children- Teacher (PSPCSA-T)	Harter, Pike [62]	–	–	–	*Convergent* Subscale correlations with Pictorial Scale of Perceived Competence and Social Acceptance for Young Children (child version) were *r* = 0.37, *p* < 0.001 (Cognitive), *r* = 0.30, *p* < 0.005 (Physical competence) and *r* = 0.06 (Social acceptance) (−)	–	–	Construct validity − Internal consistency +
	Strein, Simonson [63]	–	–	–	*Convergent* Subscale correlations with Pictorial Scale of Perceived Competence and Social Acceptance for Young Children (child version) were *r* = 0.40 (cognitive) and *r* = 0.20 (Physical competence) (−)	Cronbach’s α 0.81 (Cognitive competence); Cronbach’s α 0.76 (Physical competence) Cronbach’s α 0.80 (Peer acceptance) (+)	–	
	Garrison et al. [64]	–	–	–	*Convergent* Subscale correlations with Pictorial Scale of Perceived Competence and Social Acceptance for Young Children (child version) were *r* = 0.53, *p* < 0.001 (Cognitive), *r* = 0.03 (Physical competence) and *r* = −0.09 (Peer acceptance) (−)	–	–	
Social-Emotional Assets and Resilience Scale, Teacher rating form (SEARS-T)	Merrell et al. [65]	EFA: 4 factors explaining for 63.96% of total variance. CFA: χ^2^ (2) = 9.78, *p* = 0.01; CFI = 0.997; RMSEA = 0.068; SRMR = 0.010 (+)	–	–	*Convergent* Correlations with Social Skills Rating Scale *r* = 0.82 and Peer Relations subscale of the School Social Behavior Scale-2 *r* = 0.90 (2+) *Known-groups* Gender: Girls scored higher than boys on all factors and total score (*t* = 7.31, *p* < 0.05, Cohen’s d ES = 0.36) Special education status: children without disabilities or those not receiving special education services scored higher than those receiving special education services (*t* = − 11.76, *p* < 0.05, Cohen’s d ES = 0.74) Grade: Students in primary and elementary grades (K-6) scored higher than secondary grades (7–12) on total SEARS-T score. Differences non-significant. (*t* = 1.19, Cohen’s d ES = 0.05) Teacher-perceived levels of academic performance: Lower perceived levels of academic performance associated with lower mean SEARS-T scores and vice versa (*p* < 0.001). Cohen’s d ES = 0.51–2.04. Ethnicity: Non-significant differences in SEARS-T scores (5?)	Cronbach’s α 0.95 (Responsibility); Cronbach’s α 0.94 (Social Competence); Cronbach’s α 0.95 (Self-regulation); Cronbach’s α 0.92 (Empathy); Cronbach’s α 0.98 (Total scale) (+)	–	Structural validity ± Construct validity ? Reliability ? Internal consistency ±
	Romer, Merrell [66]	–	–	–	–	–	*Test-retest* *r* = 0.94 (total); *r* = 0.90 (Self-regulation); *r* = 0.92 (Social competence); *r* = 0.84 (Empathy); *r* = 0.92 (Responsibility) (?)	
	Figueiredo et al. [67]	CFA: 40 items used. χ^2^ (732) = 1.87, *p* = 0.00; CFI = 0.92; TLI = 0.91 RMSEA = 0.06 (−)			*Convergent* Subscale correlations with the Social Skills Rating System *r* = 0.62 to 0.76, *p* < 0.001 (+) *Known-groups* Gender: Girls scored higher than boys on subscales Responsibility, Empathy, Self-Regulation and total score; *t*(233) = − 2.35, *p =* 0.02, *g* = 0.31 Age: Differences in SEARS-T total score based on age; *F*(7,227) = 2.33, *p* = .026, η^2^ = 0.06 (2?)	Cronbach’s α 0.94 (Responsibility); Cronbach’s α 0.92 (Social Competence); Cronbach’s α 0.95 (Self-regulation); Cronbach’s α 0.92 (Empathy); Cronbach’s α 0.98 (Total scale) (?)		
Social Skills Improvement System Social Emotional Learning Edition Rating Forms (SSIS SEL RF) – Teacher version	Frank Gresham et al. [68]	CFA: Six factor model χ^2^ = 11.225, *p* < 0.0001; CFI = 0.75; RMSEA = 0.08 (90% CI = 0.079, 0.82) (−)				For ages 5–12 years, Cronbach’s α 0.96 (total), Cronbach’s α 0.78 (Self-Awareness); Cronbach’s α 0.91 (Self-Management); Cronbach’s α 0.91 (Social Awareness); Cronbach’s α 0.90 (Relationship Skills); Cronbach’s α 0.80 (Responsible Decision-Making); Cronbach’s α 0.97 and (Academic Competence) (?)	*Test-retest* *r* = 0.84 (total) *Interrater* Agreement between two teacher ratings *r* = 0.69 (2?)	Structural validity − Internal consistency ? Test-retest ?
Teacher-Child Rating Scale (T-CRS)	Hightower et al. [69]	PCA: 3 factors for problem behaviours accounted for 75.6% of the total variance. 3 factors found for competence accounting for 74.6% of total variance (?)	–	–	*Convergent* Correlations with Classroom Adjustment Rating Scale subscales *r* = 0.72 to 0.89 Correlations with Health Resources Inventory *r* = 0.56 to 0.82 (2+) *Known-groups* Program/No program comparison: program sample was rated more maladjusted/less competent than the no-program sample (*p* < 0.0001) Location, Sex, Grade: Urban children had significantly more problems and fewer competencies than suburban children. Boys had significantly higher Acting out and Learning problem scores, whereas girls had significantly higher Task Orientation scores and directionally higher Frustration Tolerance scores. No significant grade effects or interactions (4?)	Cronbach’s alphas ranged from 0.85 to 0.95 for samples A and B (+)	*Test-retest* 10 and 20-week test-retest coefficients ranged from 0.61 to 0.91 (?)	Structural validity ? Construct validity ? Internal consistency + Reliability ?
Teacher Questionnaire (TQ)	Jensen et al. [70]	–	–	–	*Convergent* Subscales phi correlations with Primary Self Concept Scale were − 0.04 to 0.57 at initial testing and − 0.05 to 0.33 at retest (−)	–	–	Construct validity −
Teacher Rating of Social Efficacy	Wheeler, Ladd [71]	EFA: Two factors accounted for 70% of the total variance (?)	–	–	*Convergent* Correlations with the Children’s Self-Efficacy for Peer Interaction Scale *r* = 0.67, *p* < 0.01 for Indiana sample and *r* = 0.29, *p* < 0.01 for New York sample (±)	Cronbach’s α 0.73 (?)	*Test-retest* *r* = 0.96 (third grade); *r* = 0.97 (fourth grade); and *r* = 0.95 (fifth grade) (?)	Structural validity ? Construct validity ± Reliability ? Internal Consistency ?
Winnetka Scale for Rating School Behaviour	Van Alstyne [72]	Three factors found using the Thurstone method (?)	–	–	*Convergent* Correlation with Schedule A, Behaviour Problem Record *r* = 0.54 and with Schedule B, Behaviour Rating Scale *r* = 0.68. Correlations with the Emotional and Social Divisions of the Haggerty-Olson Scale *r* = 0.71 (2+)	–	0.87 for the entire scale (?)	Structural validity ? Construct validity + Reliability ?
	Leton et al. [73]	PCA: Six factor model with factor loadings for total group for Responsibility 79%, Cooperation, Leadership, Emotional independence (?)	–	–	*Known-groups* Gender: Girls rated higher than boys for cooperation scales and directing group tasks. Boys received higher mean ratings for Independence of Adult Approval and Self-confidence (?)	–	–	
**Tri-Domain Measures**								
Children Activity Scales for Teachers (CHAS-T)	Rosenblum [74]	EFA: 3 factors accounting for 68% of total variance (?)	With Movement Assessment Battery for Children test *r* = 0.75, *p* < 0.0001 (+)	–	*Convergent* Correlation with Children Activity Scales for Parents (*r* = 0.59, *p* < 0.001) (+) *Discriminant* Significant differences found between two groups (i.e. TD and DCD) (*t* = 4.36, *df* = 49, *p* < 0.0001) (?)	Cronbach’s α 0.96 (?)	–	Content validity *Relevance* ± *Comprehensiveness* ± *Comprehensibility* ± Structural validity ? Criterion validity + Construct validity ± Internal consistency ?
Gross Motor Rating Scale (GMRS)	Netelenbos [75]	2 factors accounting for 73.5% of the total variance (?)	With Movement Assessment Battery for Children test *r* = 0.29 (n.s) (−)	–	*Convergent* Correlations with stepping-stone motor test (crossing time) (*r* = − 0.32, *p* < 0.01); and Test of Gross Motor Development-locomotor subtest (*r* = − 0.41, *p* < 0.01) (2−)	Cronbach’s α 0.98 (?)	*Test–retest* Class A: *r* = 0.90 (*N* = 27), Class B: *r* = 0.91 (*N* = 32); Class C: *r* = 0.88 (*N* = 23) *Interrater* *r* = 0.79 (?)	Structural validity ? Criterion validity − Construct validity − Reliability ? Internal consistency ?
Harter’s Teacher’s Rating Scale of Children’s Actual Behaviour (TRS)	Cole et al. [76]	–	–	–	*Convergent* Inter-battery factor analyses extracted one factor for third-grade boys and third-grade girls, three factors for sixth-grade boys, and two factors for sixth-grade girls. All of the factors had strong correspondence between teachers and self-ratings. Teacher ratings of a particular domain loaded onto the same factor as self-ratings of the same domain (?)	–	–	Structural validity + Construct validity ?
	Cole et al. [77]	χ^2^ = 684.58 (*df* = 395), GFI = 0.91, CFI = 0.97, RMSEA = 0.033, *p* > 0.99 (+)			*Convergent* Evident in significant trait factor loadings χ^2^ (230, *N* = 495) = 382.52, GFI = 91, CFI = 95, RMSEA = 0.054. Factor loadings on the TRS compared with the PRS. Factor loadings not significantly different for the subscales: Academic competence, Social Acceptance, Athletic competence and Behavioural conduct, with the exception of Physical Appearance subscale (?) *Discriminant* χ^2^ (230, *N* = 495) = 382.52, GFI = 91, CFI = 95, RMSEA = 0.054. The multigroup model fit the data model without allowing scales to cross-load (?)	–	–	
	Cole et al. [78]	–	–	–	*Convergent* Evident in the size and significance of appropriate factor loadings. Measures loaded onto their respective factor (*p* < 0.001) (?) *Discriminant* Model was a good fit for the data. 1 pair of constructs (out of 10 possible pairs) appeared to lack discriminant validity: social acceptance and physical appearance (?)	–	–	
Health Resources Inventory (HRI)	Gesten [79]	EFA: Five factor model accounting for 71% of total variance (?)	–	–	*Convergent* Correlation with Classroom Activity Rating Scale *r* = − 0.80 (+) *Known groups* Residence: County children had significantly higher scores than city children except on Frustration Tolerance subscale. Sex: Girls had significantly higher HRI scores than boys except on Gutsy subscale. Grade: Older children had higher HRI scores, but only Gutsy subscale showed significant grade difference (3?) *Discriminant* Mean score for normal and disturbed samples compared showed that the normative sample had significantly higher HRI scores *t*(df) = 6.28, *p* < 0.001 (?)	–	*Test-retest* *rs* = 0.87 (total scale), *rs* = 0.83 (Good student), *rs* = 0.77 (Gutsy), *rs* = 0.72 (Peer Sociability), *rs* = 0.91 (rules), and *rs* = 0.87 (Frustration Tolerance) (?)	Structural validity ? Construct validity ? Reliability ?
Social and Emotional Competencies Evaluation Questionnaire Teacher’s version – Short Form (QACSE-P-SF)	Coelho et al. [80]	CFA: χ^2^ /*df* = 1.546; CFI = 0.961; GFI = 0.896; RMSEA = 0.041 (+)	–	–	*Known-groups* Gender: Higher values for Self-Control, *t*(328) = 6.71, *p* < 0.001, Social Awareness, *t*(311) = 3.24, *p* = 0.001, and Relationship Skills, *t*(328) = 1.98, *p* < 0.05 were attributed to girls. Grade: Significant differences were found in Social Awareness, *F*(2,327) = 5.71, *p* < 0.005, Relationship Skills, *F*(2, 327) = 4.42, *p* < 0.05, and Responsible Decision Making, *F*(4,325) = 8.17, *p* < 0.001. First cycle students had higher Social Awareness and Relationship Skills than third-cycle students, while second-cycle students had higher scores than first- and third-cycle students in Responsible Decision Making (2?)	Cronbach’s α 0.84 (Self-control); Cronbach’s α 0.81 (Social Awareness); Cronbach’s α 0.92 (Relationship Skills); Cronbach’s α 0.91 (Social Isolation), Cronbach’s α 0.84 (Social Anxiety); Cronbach’s α 0.85 (Responsible Decision Making) (+)	*Test-retest* *r* = 0.74 (Self-control); *r* = 0.68 (Social Awareness); *r* = 0.69 (Relationship Skills); *r* = 0.66 (Social Isolation), *r* = 0.73 (Social Anxiety); *r* = 0.57 (Responsible Decision Making) (?)	Structural validity + Construct validity ? Reliability ? Internal consistency +
Social Skills Rating Scale (SSRS-T)	Clark et al. [81]	EFA: 4 factors accounted for 57% of total variance (?)	–	–	*Convergent* Correlation with Teacher rating of Academic Performance items (*r* = 0.60–0.64; *p* < 0.0001) Correlation with Walker Problem Behavior Identification Checklist (*r* = − 0.54; *p* < 0.01) (2−)	Cronbach’s α 0.96 (?)	–	Structural validity ? Construct validity ? Internal consistency ?
	Frank M Gresham et al. [82]	EFA: 4 factor model accounting for 45.6% of total variance (?)	–	–	*Known groups* Correlation of the variable’s student sex, student race, grade, and age with Social Skills Rating Scale ranged between *r* = − 0.11 to 0.12 (n.s). Correlations significant for Social Skills Rating Scale total score and Teachers race *r* = − 0.15, *p* < 0.05 (5?)	Cronbach’s α 0.96 (total), Cronbach’s α 0.93 (Academic Performance); Cronbach’s α 0.89 (Social Initiation); Cronbach’s α 0.92 (Cooperation); Cronbach’s α 0.75 (Peer Reinforcement) (+)	–	Reliability ?
	Elliott et al. [83]	–	–	–	*Divergent* Correlations with Revised Behaviour Problem Checklist subscales ranged between *r* = − 0.37 and − 0.93, *p* < 0.01 No significant correlations (median *r* = 0.12) were observed with the Teacher rating of Academic Performance (2−) *Known-groups* Grade differences found (?)	Time 1 Cronbach’s α = 0.96; Time 2 Cronbach’s α 0.95 (?)	*Test-retest* *r* = 0.90 (?) *Interrater* Agreement between teacher and observer ratings *r* = 0.65, *p* < 0.05 (2?)	
Teacher Estimation of Activity Form (TEAF)	Faught et al. [17]	EFA: unifactorial, first eigenvalue = 8.0, second eigenvalue = 0.3 (?)	With Bruininks–Oseretsky test of motor proficiency-short form AUC = 0.77, 95% CI: 0.68–0.86; Sensitivity = 0.85, CI: 0.68–0.94; Specificity = 0.46, CI: 0.42–0.51 for cut-off score < 32 (+)	–	*Convergent* Correlations with the Children’s Self-perceptions of Adequacy in and Predilection toward Physical Activity questionnaire (*r* = 0.45, *p* = 0.001), Participation Questionnaire (*r* = 0.25, *p* = 0.001), VO_2max_ (*r* = 0.56, *p* = 0.001), and BMI (*r* = − 0.25, *p* = 0.001) (2+, 2−)	Cronbach’s α 0.98 (+)	–	Structural validity ? Criterion validity + Construct validity ± Internal consistency +
	Sara Rosenblum, Engel-Yeger [84]	1 factor accounting for 82.5% of the total variance (?)	With MABC test *r* = 0.76, *p* < 0.01 for DCD group; Sensitivity = 73%; Specificity = 27% (+)	–	*Known-groups* Gender: Males: Mean = 3.04 ± 0.95; Females: Mean = 3.02 ± 0.98, *t*(121) = 0.103 (n.s) TD versus DCD: TD: Mean = 3.5 ± 0.84; DCD: Mean = 2.46 ± 0.75, *t*(121) = 7.15, *p* < 0.0001 (2?)	Cronbach’s α 0.975 (+)	–	

**+** Sufficient, − insufficient, ± inconsistent,? Indeterminate, *ABIC* adjusted Bayesian information criterion, *AGFI* adjusted goodness-of-fit index, *AIC* Akaike information criterion, *AUC* Area Under Curve, *BMI* body mass index, *CFA* confirmatory factor analysis, *CFI* comparative fit index, *CI* confidence interval, *CR* construct reliability, *EFA* exploratory factor analysis, *GFI* goodness-of-fit index, *ICC* intraclass correlation coefficient, *NNFI* non-normed fit index, *n.s.* non-significant, *PCA* principal component analysis, *RMSEA* Root Mean Square Error of Approximation, *SRMR* standardized root mean square residual, *TD* typically developing, *TLI* Tucker-Lewis index, *VE* variance extracted, *VO*_*2max*_ maximum volume of oxygen, *WRMR* weighted root mean square residual

*Criterion validity*, performed for five tools, was rated as sufficient for the CHAS-T, MOQ-T and TEAF; inconsistent for the MABC-2 Checklist; and insufficient for the GMRS. *Cross-cultural validity* was evaluated for the MABC-2 Checklist and was rated as indeterminate because no multiple group factor analysis was performed in the single study. For *construct validity*, results were mostly indeterminate in rating. *Internal consistency* coefficients were sometimes provided for the entire scale and/or its subscales. For the most part, tools were rated as indeterminate as a result of insufficient evidence on structural validity and/or provision of Cronbach alpha values for the total scale and not per subscale. Results quality for *test-retest and inter-rater reliability* were mostly indeterminate as intraclass correlation coefficient (ICC) values were not calculated for continuous scores. The only exception was the TRS Checklist which had ICC values for most subscales less than 0.70 and was considered as having insufficient reliability. Overall, no tool was consistently evaluated as having sufficient ratings for all its measurement properties. Only five tools (i.e. MOQ-T, ERC, MASCS, QACSE-P-SF, and TEAF) had atleast two sufficient ratings across its measurement properties.

#### Physical literacy alignment

Item/content alignment of each tool with the APLF was appraised (see Table 4). Also highlighted in Table 4 are tools with good methodological and sufficient results (i.e. atleast two sufficient ratings) quality based on evidence synthesis; as well as tools (*n* = 10) assessing the PL elements in the context of physical activity. The number of measures that mapped onto individual APLF elements ranged from 1 to 15. All elements in three (i.e. the physical, psychological, and social) out of four domains of the framework were addressed. *Relationships*, *self-regulation (emotions)*, and *collaboration* were the elements most frequently assessed by the included measures. Least captured elements were *speed*, *connection to place*, and *tactics*. Water skills, a component of the element *movement skills*, was assessed in one tool [53]. Four of the APLF elements belonging to the cognitive domain (*content knowledge*, *reasoning*, *strategy and planning*, and *perceptual awareness*) were not addressed by any measure.

**Table 4:** An overall indication of the quality of each instrument and alignment with the APLF elements

Instrument name	Evidence of good methodological quality	Evidence of sufficient results quality	Item(s) alignment with the APLF																														Total score for elements assessed (/30)
			1	2	3	4	5	6	7	8	9	10	11	12	13	14	15	16	17	18	19	20	21	22	23	24	25	26	27	28	29	30	
			Movement skills	Moving using equipment	Object manipulation	Coordination	Stability/balance	Flexibility	Agility	Strength	Muscular Endurance	Cardiovascular endurance	Reaction time	Speed	Engagement and enjoyment	Confidence	Motivation	Connection to place	Self-perception	Self-regulation (emotions)	Self-regulation (physical)	Relationships	Collaboration	Ethics	Society and culture	Content knowledge	Safety and risk	Rules	Reasoning	Strategy and planning	Tactics	Perceptual awareness	
**Single domain measures**																																	
Motor Observation Questionnaire for Teachers (MOQ-T)^a^	**✓**	**✓**	**•**	**•**	**✓**	**✓**	**✓**	**✓**	**✓**	**•**	**•**	**•**	**✓**	**•**	**•**	**•**	**•**	**•**	**•**	**•**	**•**	**•**	**•**	**•**	**•**	**•**	**•**	**•**	**•**	**•**	**•**	**•**	6
Movement Assessment Battery for Children-2 Checklist (MABC-2 Checklist)^a^	**✓**	✘	**✓**	**✓**	**✓**	**✓**	**✓**	**•**	**•**	**•**	**•**	**•**	**•**	**•**	**•**	**•**	**•**	**•**	**•**	**•**	**•**	**•**	**•**	**•**	**•**	**•**	**•**	**•**	**•**	**•**	**•**	**•**	5
Pictorial Scale of Perceived Water Competence (PSPWC)^a^	**✓**	✘	**✓**	**•**	**•**	**•**	**•**	**•**	**•**	**•**	**•**	**•**	**•**	**•**	**•**	**•**	**•**	**•**	**•**	**•**	**•**	**•**	**•**	**•**	**•**	**•**	**•**	**•**	**•**	**•**	**•**	**•**	1
Reiss Motivation Profile for children (Child RMP)^a^	**✓**	✘	**•**	**•**	**•**	**•**	**•**	**•**	**•**	**•**	**•**	**•**	**•**	**•**	**•**	**•**	**✓**	**•**	**•**	**•**	**•**	**•**	**•**	**•**	**•**	**•**	**•**	**•**	**•**	**•**	**•**	**•**	1
Teacher’s Self-concept Evaluation Scale	✘	✘	**•**	**•**	**•**	**•**	**•**	**•**	**•**	**•**	**•**	**•**	**•**	**•**	**•**	**•**	**•**	**•**	**✓**	**•**	**•**	**•**	**•**	**•**	**•**	**•**	**•**	**•**	**•**	**•**	**•**	**•**	1
Teen Risk Screen checklist (TRS)^a^	**✓**	✘	**✓**	**•**	**✓**	**•**	**✓**	**•**	**•**	**•**	**•**	**•**	**•**	**•**	**•**	**•**	**•**	**•**	**•**	**•**	**•**	**•**	**•**	**•**	**•**	**•**	**•**	**•**	**•**	**•**	**•**	**•**	3
**Dual-domain measures**																																	
Brief Behaviour Rating Scale (BBRS)	✘	✘	**•**	**•**	**•**	**•**	**•**	**•**	**•**	**•**	**•**	**•**	**•**	**•**	**•**	**•**	**•**	**•**	**•**	**✓**	**•**	**✓**	**✓**	**•**	**•**	**•**	**•**	**•**	**•**	**•**	**•**	**•**	3
Devereux Student Strengths Assessment (DESSA)	**✓**	✘	**•**	**•**	**•**	**•**	**•**	**•**	**•**	**•**	**•**	**•**	**•**	**•**	**•**	**•**	**•**	**•**	**✓**	**✓**	**✓**	**✓**	**✓**	**•**	**•**	**•**	**•**	**•**	**•**	**•**	**•**	**•**	5
Emotion Regulation Checklist (ERC)	✘	**✓**	**•**	**•**	**•**	**•**	**•**	**•**	**•**	**•**	**•**	**•**	**•**	**•**	**•**	**•**	**•**	**•**	**•**	**✓**	**•**	**✓**	**•**	**•**	**•**	**•**	**•**	**•**	**•**	**•**	**•**	**•**	2
Multisource Assessment of Social Competence Scale (MASCS)	**✓**	**✓**	**•**	**•**	**•**	**•**	**•**	**•**	**•**	**•**	**•**	**•**	**•**	**•**	**•**	**•**	**•**	**•**	**•**	**✓**	**•**	**✓**	**✓**	**•**	**•**	**•**	**•**	**•**	**•**	**•**	**•**	**•**	3
Pictorial Scale of Perceived Competence and Social Acceptance for Young Children-Teacher (PSPCSA-T)^a^	**✓**	✘	**✓**	**•**	**✓**	**•**	**•**	**•**	**•**	**•**	**•**	**•**	**•**	**•**	**•**	**•**	**•**	**•**	**•**	**•**	**•**	**✓**	**•**	**•**	**•**	**•**	**•**	**•**	**•**	**•**	**•**	**•**	3
Social-Emotional Assets and Resilience Scale, Teacher rating form (SEARS-T)	**✓**	✘	**•**	**•**	**•**	**•**	**•**	**•**	**•**	**•**	**•**	**•**	**•**	**•**	**•**	**•**	**•**	**•**	**•**	**✓**	**•**	**✓**	**✓**	**•**	**•**	**•**	**•**	**•**	**•**	**•**	**•**	**•**	3
Social Skills Improvement System Social Emotional Learning Edition Rating Forms (SSIS SEL RF) – Teacher version	**✓**	✘	**•**	**•**	**•**	**•**	**•**	**•**	**•**	**•**	**•**	**•**	**•**	**•**	**•**	**•**	**•**	**•**	**✓**	**•**	**✓**	**✓**	**•**	**✓**	**•**	**•**	**•**	**•**	**•**	**•**	**•**	**•**	4
Teacher-Child Rating Scale (T-CRS)	**✓**	✘	**•**	**•**	**•**	**•**	**•**	**•**	**•**	**•**	**•**	**•**	**•**	**•**	**•**	**•**	**•**	**•**	**•**	**✓**	**•**	**✓**	**✓**	**✓**	**•**	**•**	**•**	**•**	**•**	**•**	**•**	**•**	4
Teacher Questionnaire (TQ)	✘	✘	**•**	**•**	**•**	**•**	**•**	**•**	**•**	**•**	**•**	**•**	**•**	**•**	**•**	**•**	**•**	**•**	**✓**	**✓**	**•**	**✓**	**✓**	**•**	**•**	**•**	**•**	**•**	**•**	**•**	**•**	**•**	4
Teacher Rating of Social Efficacy	✘	✘	**•**	**•**	**•**	**•**	**•**	**•**	**•**	**•**	**•**	**•**	**•**	**•**	**•**	**•**	**•**	**•**	**•**	**•**	**•**	**✓**	**✓**	**•**	**•**	**•**	**•**	**✓**	**•**	**•**	**•**	**•**	3
Winnetka Scale for Rating School Behaviour	✘	✘	**•**	**•**	**•**	**•**	**•**	**•**	**•**	**•**	**•**	**•**	**•**	**•**	**•**	**•**	**•**	**•**	**•**	**✓**	**✓**	**✓**	**✓**	**•**	**•**	**•**	**•**	**•**	**•**	**•**	**•**	**•**	4
**Tri-domain measures**																																	
Children Activity Scales for Teachers (CHAS-T)^a^	✘	✘	**✓**	**•**	**✓**	**•**	**✓**	**•**	**•**	**•**	**•**	**•**	**•**	**•**	**•**	**•**	**•**	**•**	**•**	**•**	**✓**	**•**	**•**	**•**	**•**	**•**	**•**	**✓**	**•**	**•**	**•**	**•**	5
Gross Motor Rating Scale (GMRS)^a^	✘	✘	**✓**	**✓**	**✓**	**✓**	**✓**	**✓**	**✓**	**✓**	**✓**	**✓**	**✓**	**✓**	**✓**	**•**	**✓**	**•**	**•**	**•**	**•**	**•**	**•**	**•**	**•**	**•**	**✓**	**•**	**•**	**•**	**•**	**•**	15
Harter’s Teacher’s Rating Scale of Child’s Actual Behaviour (TRS)^a^	**✓**	✘	**•**	**•**	**•**	**•**	**•**	**•**	**•**	**•**	**•**	**•**	**•**	**•**	**•**	**•**	**•**	**•**	**•**	**•**	**•**	**✓**	**•**	**•**	**•**	**•**	**•**	**✓**	**•**	**•**	**•**	**•**	–
Health Resources Inventory (HRI)	✘	✘	**•**	**•**	**•**	**•**	**•**	**•**	**•**	**•**	**•**	**•**	**•**	**•**	**•**	**✓**	**•**	**✓**	**✓**	**✓**	**•**	**✓**	**✓**	**✓**	**✓**	**•**	**•**	**✓**	**•**	**•**	**•**	**•**	9
Social and Emotional Competencies Evaluation Questionnaire Teacher’s version – Short Form (QACSE-P-SF)	**✓**	**✓**	**•**	**•**	**•**	**•**	**•**	**•**	**•**	**•**	**•**	**•**	**•**	**•**	**•**	**•**	**•**	**•**	**•**	**✓**	**•**	**✓**	**✓**	**✓**	**•**	**•**	**✓**	**•**	**•**	**•**	**•**	**•**	5
Social Skills Rating Scale (SSRS-T)	**✓**	✘	**•**	**•**	**•**	**•**	**•**	**•**	**•**	**•**	**•**	**•**	**•**	**•**	**•**	**•**	**•**	**•**	**•**	**✓**	**•**	**✓**	**✓**	**✓**	**✓**	**•**	**•**	**✓**	**•**	**•**	**•**	**•**	6
Teacher Estimation of Activity Form (TEAF)^a^	**✓**	**✓**	**•**	**•**	**•**	**•**	**•**	**•**	**✓**	**✓**	**✓**	**✓**	**•**	**•**	**✓**	**✓**	**✓**	**•**	**•**	**•**	**•**	**•**	**•**	**•**	**•**	**•**	**•**	**•**	**•**	**•**	**✓**	**•**	8
**Total tools addressing each element of the APLF**	**15**	**5**	**6**	**2**	**6**	**3**	**5**	**2**	**3**	**2**	**2**	**2**	**2**	**1**	**2**	**2**	**3**	**1**	**5**	**11**	**4**	**15**	**11**	**5**	**2**	**0**	**2**	**5**	**0**	**0**	**1**	**0**	

**✓** Elements assessed, evidence of good methodological quality or sufficient results quality, **•** elements not assessed, ✘ absence of good methodological quality or sufficient results quality, ^a^Tools contain items that assess elements in the context of physical activity

Tools capturing the most elements of the APLF included the GMRS (15 out of 30), the HRI (9 out of 30), and the TEAF (8 out of 30). Lastly, Harter’s TRS covered three domains (physical, social, cognitive) of the framework. However, due to the lack of specificity of items contained within the tool (e.g., “This child doesn’t do well at new outdoor games”; “This child does really well at all kinds of sports”; “This child is better than others his/her age at sports”), mapping it onto the individual elements of the framework proved rather difficult.

## Discussion

This is the first review to critically evaluate the psychometric properties of teacher proxy-report instruments designed to assess one or more elements of children’s PL. As a consequence, the current study represents a novel contribution to the literature base relating to PL and its assessment. PL assessment can help identify aspects of children’s PL that are suboptimal; as well as provide an evidence base for evaluating the effectiveness of interventions targeted at improving PL levels. More specifically, a focus on teacher proxy-report instruments for children’s PL is needed due to children’s limited cognitive abilities when making self-assessments of their own capabilities [27, 62, 85]. Baranowski [86] has further suggested that children are also limited in their ability to recall specific events that occurred in the past. Indeed, Bardid et al. [25] has reported that teacher proxy-reports (especially by physical education specialists) may provide more accurate estimates of a child’s capabilities (e.g., motor competence) than child self-report.

Importantly, in the current review, alignment with individual elements of the APLF, for each teacher proxy-report measure, was further appraised. The first finding is clearly the lack of valid and reliable teacher proxy-report instruments that assess PL in its entirety, based on the comprehensive APLF. There are however tools available to assess some elements of the framework. Specifically, 41 studies evaluating the psychometric properties of 24 teacher proxy-report tools for the APLF elements were identified. The psychometric properties of identified measures were variable, with many typically unreported or inadequately assessed.

### Psychometric properties

No single tool reported all nine psychometric properties outlined by the COSMIN methodology [35–37]. Measurement properties frequently reported included construct validity, structural validity, and internal consistency. Content validity and cross-cultural validity were the most rarely reported. No studies reported measurement error and responsiveness. These mirror findings of a recently published review of motor competence assessments for children and adolescents, which highlighted that construct validity was frequently reported whereas content validity was the least evaluated psychometric property [43].

Content validity is often considered the most important measurement property of an instrument [87], and is needed to ensure that the tool has appropriate number of items and adequately captures the construct/element under investigation [88]. COSMIN distinguishes between tool development and content validity studies in that the former involves concept elicitation, development, and pilot testing a new tool; whereas the latter entails testing of an existing tool [87]. In this review, most tool development studies were given the lowest possible rating of “inadequate”. This was either because tool development studies were not performed utilizing a sample representative of the tool’s targeted population or no pilot tests or cognitive interviews were performed for the newly developed tool. On the other hand, just two studies reported on content validity for the CHAS-T [74] and PSPWC [53]. The comprehensibility, relevance, and comprehensiveness of items in the CHAS-T [74] was explored by teachers and professionals. In this review, the instrument was rated as doubtful for methodological quality as there was a lack of reporting of the qualitative and analytical methods utilized for the content validation process. Another study reported content validity for the TRS tool [56]; however, the review team failed to find any report regarding the relevance or comprehensiveness of items from the perspective of the targeted users of the tool and/or professionals. The PSPWC [53] was rated as doubtful in methodological quality as it was not clear if there were two researchers involved in analysis of qualitative interviews and whether skilled interviewers were used during interviews.

According to COSMIN’s updated guidelines, if the content validity of a tool is unknown, the results for other measurement properties of the tool should be ignored and not further appraised as this hinders the interpretation and generalization of study findings [36]. Given the importance of this measurement property, there is an urgent need to prioritize content validity studies in future development of teacher proxy-report PL instruments. Future studies should consider using the COSMIN Study Design checklist [89] which offers clear standards for designing studies aimed to evaluate measurement properties of instruments. Specifically, for content validity studies, tool developers should obtain information from targeted tool users and professionals regarding the relevance, comprehensibility, and comprehensiveness of the instructions, response options and items contained within the tool. For this, a widely recognized or well justified qualitative research approach is preferred, whereby each item on the tool is evaluated by at least seven or more individuals from the target population of interest and professionals – see Mokkink et al. [89] for the design requirements.

Few studies validated a measure against a reference “gold” standard known as criterion validity. Criterion validity ensures the accuracy of a scale when compared to a reference standard [90]. Being widely tested and validated measures, the MABC motor test [91], the Bruininks–Oseretsky test of motor proficiency [92], and the Körperkoordinationstest für Kinder test [93] were considered to be reasonable “gold” standards for motor skill assessment. Hence, all studies comparing a teacher proxy-report tool to these measures were considered a study on criterion validity [36]. It is important to note that there were a few cases where authors used the term criterion validity when comparisons were made with other measures assessing a similar construct. In these instances (as specified in the COSMIN user manual [36]), this was considered to be evidence of construct validity rather than criterion validity. In this review, most studies on criterion validity appeared to have good methodological quality, with evaluated measures having sufficient results quality. Similar findings were noted by Antczak et al. [43] for criterion validity studies of motor competence assessments. However, it has been argued that the design of the COSMIN checklist, in terms of number of standards contained in each measurement property and the use of the “worst score counts” principle, could significantly impact on its overall scoring. For instance, a measurement property such as criterion validity which contains fewer standards (three in total) may fare better in its overall scoring when compared to those with higher quality items (e.g., 35 standards for content validity) [43].

The methodological quality of studies reporting structural validity was mixed. The common reasons for doubtful or inadequate COSMIN ratings were insufficient sample size and/or statistical design flaws such as a lack of reporting of the number of teachers involved in the study and how these clustering effects (if any) were accounted for in the analytical design. Furthermore, for many tools, result ratings were indeterminate due to the use of exploratory factor analysis (including principal component analysis) as the updated COSMIN does not provide any criteria for rating these techniques. Ideally, a confirmatory factor analysis should follow an exploratory factor analysis (preferably using a different sample), as the former verifies an a priori exploratory factor analysis-informed theory regarding a tool’s factor structure [94]. Given that some of these deficiencies can be resolved by more detailed reporting and further psychometric testing, future studies should consider adopting guidelines offered by COSMIN for reporting of structural validity studies.

Only one of 41 studies was assessed for cross-cultural validity, as they had translated a measure (MABC-2 Checklist) from English to Japanese, and compared scores obtained from two samples (i.e. United Kingdom and Japan) [50]. This study did not perform well for both methodological and results quality. Noteworthy is that a number of studies [47, 48] within this review translated a measure from its original language to a different language without assessing cross-cultural validity. Future studies should determine cross-cultural validity for translated instruments, utilizing appropriate techniques (e.g., multi-group confirmatory factor analysis for classical test theory or differential item functioning for item response theory) [35, 36]. This is because instruments may perform differently across different cultures, different gender or age groups, and different populations [95]. Most construct validity studies performed adequately for methodological quality; however, overall results quality was mostly indeterminate. This may have been influenced by the lack of a priori hypotheses for expected differences between groups for known groups/discriminant validity.

Internal consistency values (the interrelated among items in a subscale [36]) had to be calculated separately for each unidimensional scale or subscale to obtain good ratings for methodological quality. Deficiencies in studies were mostly because Cronbach’s alpha values were provided for the entire scale and not per subscale. Similarly, results of internal consistency were indeterminate for many studies as Cronbach alpha was provided for the entire scale and there was evidence of insufficient structural validity. COSMIN considers evidence on structural validity (or unidimensionality) a prerequisite for interpreting Cronbach’s alpha values [36]. Given these findings, we recommend that as a starting point, future studies should ensure that evidence exists for sufficient unidimensionality or structural validity of a tool and thereafter report on the Cronbach alphas (for continuous scores) of each subscale.

Reliability (test-retest and inter-rater) studies did not rate well for methodological quality for studies in this review. For the majority of studies, Pearson’s correlations (a measure of relationship between two variables [96]) were used to explore this measurement property rather than intraclass correlations for continuous scores, as recommended by the COSMIN [36]. Past literature has highlighted that the Pearson’s is an inappropriate and liberal measure of reliability, often producing reliability coefficients that are higher than the true reliability [88, 97]. It was also difficult to determine whether participants were stable in the interim between measurements or if the testing conditions were similar for the measurements taken. As ICC values were not calculated, results were rated as indeterminate for the majority of studies in this review. Studies should consider the use of intraclass correlations when exploring reliability of continuous variables as they reflect the correlation and agreement between measurements taken by an instrument [96].

Two measurement properties – responsiveness and measurement error – were not explored in any study in this review. COSMIN refers to responsiveness as the measures ability to detect change over time in the construct of interest whereas measurement error is regarded as errors in scores obtained which are not as a result of changes in the construct of interest [36]. No study included in this review evaluated the minimal important change or minimal important difference of their tools. Without information on the measurement error of these tools, it is unclear whether the changes in scores of the constructs assessed are meaningful and matter to teachers. Studies have also previously noted underreporting of responsiveness [98]. This is concerning because without this, it is difficult to assess the effectiveness of interventions designed to improve PL or its components.

In summary, for the studies included in this review, a median of 3 out of nine psychometric properties were reported. Content validity which is considered the most important property was sparingly reported. These therefore restricts our justifications for use of specific teacher proxy-report tools in practice until further psychometric testing is conducted. However, based on the available evidence and after combining the ratings of methodological quality and the criteria for good measurement properties provided by the COSMIN, best results were received for the following tools: MASCS, MOQ-T, QACSE-P-SF and TEAF. These tools combined assess a total of 18 elements of the APLF. Of these tools, the MOQ-T and TEAF assesses the APLF elements in relation to physical activity. The ERC had good psychometric evidence but was lacking in methodological rigour. Terwee et al. [99] has highlighted that results of studies lacking in methodological quality should not be trusted. One must exercise caution when interpreting these results though as some of these tools (specifically MASCS and QACSE-P-SF) were evaluated in single studies, and as such, are in need of repeated psychometric testing in different populations. Furthermore, in the current review, the MABC-2 checklist was found to be one of the most widely examined tool for reliability and validity. Surprisingly, despite having good methodological quality for most of its measurement properties, our findings reveal that the checklist has limited psychometric evidence to support its reliability and validity, suggesting the need for more validation studies. The current systematic review highlights a need for further psychometric testing (especially content validity, cross-cultural validity, measurement error, criterion validity, and responsiveness), with a more detailed reporting of methodological aspects and results in future studies. Taking such an approach will provide teachers with a more robust foundation when selecting appropriate and psychometrically sound measures for assessing PL.

### Physical literacy alignment

The APLF is unique in that it recognizes a variety of skills and attributes straddling four inter-related learning domains (physical, psychological, social, and cognitive) as needed for PL development. More specifically, the framework incorporates elements outside the physical domain that have not previously featured in other definitions. These elements may be equally beneficial for integrated movement experiences to develop PL [40]. An example element *collaboration*, situated in the social domain, reflects social skills (e.g., conflict resolution, cooperation, and leadership) required to successfully interact with others in movement and physical activity contexts [30]. This element is potentially as important as other elements (e.g., movement skills) and should be assessed in children.

Our review findings suggest the paucity of teacher proxy-report measures that address several elements of the APLF. Particularly elements such as *speed*, *connection to place*, *tactics*, *content knowledge*, *reasoning*, *strategy and planning*, and *perceptual awareness* were either rarely assessed or not assessed by identified tools. Interestingly, elements most frequently assessed appeared to fall within the social domain suggesting the availability of many teacher assessment options for this domain. Because of our wider search for tools beyond the physical activity/physical education literature, only the PSPCSA-T and Harter’s TRS assessed the social domain in the context of physical activity. Our findings may be an indication that the social domain – despite not being recognized as a core component of several PL frameworks – is an aspect that teachers are interested in reporting on more generally.

Another finding is the absence of measures with psychometric evidence that address elements of the cognitive domain. The authors note however that it may be quite challenging to assess the cognitive domain via teacher proxy-reporting. Indeed, many existing measures for PL (e.g., CAPL) tend to approach its assessment via self-report [31]. Nonetheless, a comprehensive approach to assessing PL is required since the flavour of the concept in itself lies in its holistic nature [100]. Hence, the development of measures that target all domains and elements of the APLF should be prioritized to provide a greater breadth and depth of understanding of the contributors to children’s PL.

### Recommendations for teacher assessment of physical literacy based on the APLF

Proxy-report measures have the advantage of low cost, ease of administration on large numbers of children, and less administration training when compared to objective measures [25]. This is even more beneficial to teachers who are often faced with time barriers to teaching and assessment [101]. In making recommendations for teachers when choosing instruments for PL assessment, besides highlighting psychometrically sound measures, many aspects of the feasibility of these measures should be well considered. Some of these feasibility aspects include completion time, cost of instrument, copyright, length of the instrument, ease of administration and score calculation [36]. Information on feasibility may become particularly relevant when differentiating between two equally psychometrically sound instruments. The vast majority of measures identified in this review did not report on completion time. However, as feasibility is not considered a measurement property by the COSMIN [36], it was beyond the scope of this paper to consider all aspects of the feasibility of the identified tools. We therefore recommend that these aspects receive priority in future studies.

As earlier stated, the current review did not locate a tool that captured all elements and domains of the APLF. For teachers to assess PL comprehensively, there is a need for a tool that includes all 30 elements of the framework. Also given limited evidence found for measures in this review, it is difficult to justify the use of tools identified in this review until further psychometric testing is conducted. This review has found best evidence for the MASCS, MOQ-T, QACSE-P-SF and TEAF. Teachers who are interested in assessing elements of PL based on its Australian approach could consider utilizing the detailed nine-step decision-making steps in choosing a PL assessment as highlighted by Barnett et al. [33], in conjunction with Tables 2, 3 and 4 of this review which provide information on the validity, reliability, and alignment of specific instruments with the APLF. Barnett et al.’s [33] guidance for assessing PL involve identifying the following: (i) element(s) of interest; (ii) teacher interest; (iii) context; (iv) purpose; (v) age group; (vi) structure of observed learning outcomes level; (vii) measurement/assessment method; (viii) number of participants and; (ix) cost. Specifically, step seven encourages teachers to decide on their preferred assessment approach (e.g., objective or subjective measures). As an example, after carefully considering these nine steps in conjunction with the results provided in Tables 2, 3 and 4, a teacher who may be interested in assessing the APLF elements *agility*, *strength*, *muscular endurance*, *cardiovascular endurance*, *engagement and enjoyment*, *confidence*, *motivation* and *tactics* (Step I) via proxy-reporting (Step VII), could utilize the TEAF. This is because, based on the available psychometric evidence (methodological quality and results quality), the tool seems to be the most promising teacher tool for assessing these aforementioned elements. An assessment of this nature by physical educators must be approached with caution, as most tools identified within this review were not contextualized in physical activity (as outlined in Table 4). As such, we have highlighted the tools assessing the PL elements in the context of physical activity – refer to Table 4.

### Strengths and limitations

This systematic review has several strengths. The protocol for the review was registered prospectively. A comprehensive search of seven databases relevant to Sport, Education, Psychology and Health was conducted to identify peer-review articles. Furthermore, a comprehensive search strategy comprising of search filters for finding studies on measurement properties provided by COSMIN; as well as search filters relevant to each individual PL element was utilized to locate studies within the review. Time restrictions were not applied in the search strategy. This strategy identified studies focused on psychometric testing of tools for each PL element, unlike previous reviews which were focused mostly on tools for PL as a whole without critically appraising the psychometric properties of those tools. Three authors were independently involved in the full-text review phase and methodological quality assessment of included studies following best practice recommendations when conducting systematic reviews. This triangulation approach reduces the risk of non-detection of relevant evidence, thus strengthening the validity of conclusions reached from available evidence [102]. Lastly, within the PL research area, this is the first systematic review performed in accordance with PRISMA guidance [34] and COSMIN’s latest 2018 guidance [35–37], which is more detailed than its 2010 guidance [103, 104].

This study is not without limitations. Only studies published in English Language were included, due to our limited resources, time and expertise in non-English languages. Studies with English abstracts and non-English full text were also excluded because when it is not possible to obtain a translation, extracting all the information needed to meaningfully inform the systematic review based on the abstract only is difficult. Therefore, some findings may have been overlooked. Furthermore, because of the lack of rigorous peer-review, grey literature including conference, poster abstracts, dissertations, and tool manuals were excluded. As such, it is possible that some measurement properties (e.g., content validity) were reported within tool manuals. Only studies reporting on one or more measurement properties outlined by the COSMIN for teacher tools of the PL elements were included in the review. Hence, a number of studies may have been omitted if measurement properties were not discussed for tools utilized in those studies. The COSMIN methodology does not differentiate between poor reporting and poor quality in the risk of bias analyses. Therefore, there could have be cases where a lack of detailed reporting by authors resulted in an inadequate or doubtful rating for methodological quality. Finally, there were tools which had multiple validity and reliability studies which shows a more widespread use. There were also instruments evaluated in a single study. This may have impacted on the overall ratings of results quality for the tools identified within this review.

## Conclusions

This review is the first to identify and critically appraise the psychometric properties of 24 teacher proxy-report measures for assessing a comprehensive framework of PL, for children aged 5–12 years. Teacher proxy-report may provide more reliable estimates of a child’s ability compared to self-report, are low in cost, and can be used to assess large sample sizes compared to objective measures. Moreover, objective assessment may not be conducive for some elements (e.g., relationships, ethics) of the APLF. Our review findings suggest that presently, there is no existing teacher proxy-report tool to assess all elements of children’s PL identified in the APLF. Based on the findings of this review, there remain considerable gaps in knowledge in aspects related to the validity (e.g., content, cross-cultural), reliability (measurement error), and responsiveness of teacher tools. This emphasizes the need for further psychometric studies on existing teacher report tools; and more importantly, the need to develop new teacher tools for assessing the PL domains in its entirety. Tool developers may consider combining items from existing scales, preferably those that have undergone repeated processes of psychometric testing for validity and reliability as highlighted in this review. As Streiner et al. [88] puts it simply “instruments rarely spring fully grown from the brows of their developers. Rather, they are usually based on what other people have deemed to be relevant, important, or discriminating”. Due to the comprehensive nature, this review raises the importance and need for a proxy-report scale for teachers within the Australian context; and teachers globally who are interested in the assessing children’s PL based on the comprehensive APLF.

## Supplementary Information

**Additional file 1.** PRISMA Checklist.

**Additional file 2.** List of search terms using Boolean connectors “AND” or “OR” to retrieve articles from the databases.

**Additional file 3.** PICO-based (Population, Intervention, Comparison, Outcome) taxonomy of reasons used to exclude articles from the systematic review.

## Data Availability

Not applicable.

## Abbreviations

APLF: Australian Physical Literacy Framework
BBRS: Brief Behaviour Rating Scale
CAMSA: Canadian Agility and Movement Skill Assessment
CAPL: Canadian Assessment of Physical Literacy
CFA: Confirmatory factor analysis
CFI: Comparative fit index
CHAS-T: Children Activity Scales for Teachers
Child RMP: Reiss Motivation Profile for children
COSMIN: COnsensus-based Standards for the selection of health Measurement Instruments
DCD: Developmental coordination disorder
DESSA: Devereux Student Strengths Assessment
EFA: Exploratory factor analysis
ERC: Emotion Regulation Checklist
GMRS: Gross Motor Rating Scale
Harter’s TRS: Harter’s Teacher’s Rating Scale of Child’s Actual Behaviour
HRI: Health Resources Inventory
MABC: Movement Assessment Battery for Children
MASCS: Multisource Assessment of Social Competence Scale
MOQ-T: Motor Observation Questionnaire for Teachers
PCA: principal component analysis
PL: Physical literacy
PLAY: Physical Literacy Assessment for Youth
PRISMA: Preferred Reporting Items for Systematic Reviews and Meta-Analyses
PSPCSA-T: Pictorial Scale of Perceived Competence and Social Acceptance for Young Children-Teacher
PSPWC: Pictorial Scale of Perceived Water Competence
QACSE-P-SF: Social and Emotional Competencies Evaluation Questionnaire Teacher’s version (Short Form)
RMSEA: Root Mean Square Error of Approximation
SEARS-T: Social-Emotional Assets and Resilience Scale, Teacher rating form
SRMR: Standardized Root Mean Residuals
SSIS SEL RF: Social Skills Improvement System Social Emotional Learning Edition Rating Forms
SSRS-T: Social Skills Rating Scale
T-CRS: Teacher-Child Rating Scale
TEAF: Teacher Estimation of Activity Form
TQ: Teacher Questionnaire
TRS: Teen Risk Screen checklist
