# Supplementary material for: A systematic review of tools designed for teacher proxy-report of children’s physical literacy or constituting elements
Source: Int J Behav Nutr Phys Act. 2021 Oct 8;18:131. doi: 10.1186/s12966-021-01162-3 (PMC8499583; doi:10.1186/s12966-021-01162-3)
Supplement: Supplementary file 2 — Additional file 2. List of search terms using Boolean connectors “AND” or “OR” to retrieve articles from the databases. [file 12966_2021_1162_MOESM2_ESM.docx]

List of search terms using Boolean connectors “AND” or “OR” to retrieve articles from the databases

| **Boolean connector** | **Search Terms** | **Limitations** |
| --- | --- | --- |
| AND | “Confirmatory factor analysis” OR “Cronbach* Alpha” OR valid* OR reliabl* OR psychometric* OR “instrument psychometric*” OR “scale analysis” OR “factor analysis” OR “test validity” OR “internal consistency” OR “criterion validity” OR “cross-cultural validity” OR “hypothes* test*” OR “content valid*” OR “face valid*” | Title, Abstract, Keyword, English Language, Peer-review |
| AND | "Self-report" OR "self-rated" OR "proxy-report" OR "proxy instrument" OR "proxy tool" OR "proxy report tool" OR "parent rated" OR "parent* report" OR "teacher rated" OR "coach* rated" OR "teacher proxy" OR "parent proxy" OR teacher OR parent OR pictorial OR picture* OR proxy | Title, Abstract, Keyword, English Language, Peer-review |
| AND | Questionnaire* OR question* OR survey* OR scale* OR instrument* OR assess* OR tool* OR measure* OR test* OR "check list" OR checklist OR screen OR inventory OR “subjective measure*” | Title, Abstract, Keyword, English Language, Peer-review |
| AND | “Primary school child*” OR “school child*” OR child* OR “young child*” OR childhood OR “elementary school child*” OR elementary OR “young person*” OR “young people” OR “5-12 year*” OR pupil* OR “young person” OR boy* OR girl* OR student* | Title, Abstract, Keyword, English Language, Peer-review |
| AND | “Motor ability” OR “movement skill*” OR “motor skill*” OR “motor development” OR “fundamental motor skill*” OR “fundamental movement skill*” OR “motor competence” OR “motor skill* competenc*” OR “movement competence” OR “motor performance” OR “gross motor ability” OR “perceived movement skill competence” OR “perceived motor skill competence” OR “motor perception*” OR “movement proficiency” OR “motor proficiency” OR “gross motor skill*” OR “gross motor development” OR “locomotor skill” OR “water skill*” OR “diving skill*” OR “swimming skill*” OR “aquatic skill*” OR “fundamental water skill*” OR “swimming stroke*” OR floating OR diving | Title, Abstract, Keyword, English Language, Peer-review |
| AND | “Mov* on equipment” OR “mov* in equipment” OR “mov* with equipment” OR bik* OR cycl* OR scooter* OR surf* OR kayak* OR sail* OR “paddle board*” OR skiing OR snowboard* OR skating OR “object locomotion” OR “boogie board*” OR “rope climb*” OR blading | Title, Abstract, Keyword, English Language, Peer-review |
| AND | “Object control skill” OR “object control” OR “object manuipulat*” OR “manipulat* skill*” OR throw* OR catch* OR dribbl* OR kick* OR strik* | Title, Abstract, Keyword, English Language, Peer-review |
| AND | “Motor coordination” OR coordination OR “perceptual motor coordination” OR “motor function” | Title, Abstract, Keyword, English Language, Peer-review |
| AND | “Static balance*” OR balance OR “counter balance” OR “counter tension” OR “walk* on a beam” OR “beam walk*” OR stability OR “isometric balance” OR “dynamic balance” OR “static stability” OR “postural stability” OR “postural balance” OR equilibrium OR “postural control” OR “stability skill*” | Title, Abstract, Keyword, English Language, Peer-review |
| AND | "Cardiovascular endurance" OR "cardiovascular fitness" OR "cardiorespiratory endurance" OR “cardiorespiratory fitness” OR "aerobic capacity" OR "maxim* oxygen consumption" | Title, Abstract, Keyword, English Language, Peer-review |
| AND | Agility OR “reactive agility” OR "change with speed" OR dodg* OR "change of direction speed" OR "planned agility" OR "non-planned agility" | Title, Abstract, Keyword, English Language, Peer-review |
| AND | “Range of motion” OR flexibility OR flexible OR limberness OR stretch* OR "static flexibility" OR "dynamic flexibility" OR pliability | Title, Abstract, Keyword, English Language, Peer-review |
| AND | “Muscl* strength” OR “physical strength” OR “strength training” OR “muscular strength” OR “push-up*” OR “pull-up*” OR “weight-lifting” OR squat* OR lunges | Title, Abstract, Keyword, English Language, Peer-review |
| AND | "Muscular endurance" OR "strength training" OR "muscular resistance" OR “sit-up*” | Title, Abstract, Keyword, English Language, Peer-review |
| AND | "Reaction time*" OR "response time*" | Title, Abstract, Keyword, English Language, Peer-review |
| AND | Sprint OR “sprint speed” "speed of movement" OR "movement speed" OR "fitness speed" OR "physical fitness N5 speed" OR speed OR accelerat* OR decelerat* | Title, Abstract, Keyword, English Language, Peer-review |
| AND | Engagement OR enjoyment OR “physical activity N2 engagement” OR "sport* N2 engagement" OR "sport involvement" OR “physical activity involvement” OR "active participation" OR "exercise N2 enjoyment" OR "sport* N5 enjoyment" OR "physical activity N2 enjoyment" OR "PA enjoyment" OR "PE enjoyment" | Title, Abstract, Keyword, English Language, Peer-review |
| AND | “Anger control” OR self-regulat* OR "emotion* regulat*" OR "physical regulat*" OR "regulation of emotion" OR "emotion* dysregulat*" | Title, Abstract, Keyword, English Language, Peer-review |
| AND | “Self-perception” OR “self-concept” OR “selfaware*” OR "strength recognition" | Title, Abstract, Keyword, English Language, Peer-review |
| AND | Motivat* OR motive OR "self-determ*" OR SDT OR amotivat* OR "extrinsic motivation" OR "intrinsic motivation" OR “autonomous motivation” OR demotivation OR "exercise motivation" OR "PE motivation" OR "PA motivation" OR "physical activity motivation" OR “motivation for physical activity” OR “motivation for PA” OR “introjected motivation” OR “readiness for PA” | Title, Abstract, Keyword, English Language, Peer-review |
| AND | “Physical activity confidence” OR "PA confidence" OR "PE confidence" OR confidence OR "selfconfidence" OR "selfefficacy" OR "perceived competence" OR "perceived motor competence" OR "sport* confidence" OR "performance expectanc*" OR "performance expectation*" OR "movement confidence" OR "perceived ability" OR “physical activity confidence” OR “confidence for physical activity” | Title, Abstract, Keyword, English Language, Peer-review |
| AND | Connectedness OR "connect* N5 physical environment" OR "connect* N2 nature" OR "connect* N2  environment" OR "nature connectedness" OR "connectedness to nature scale" OR "engag* with natural beauty" OR "nature relatedness" OR "connect* N2 natural environment" OR "connect N2 built environment" | Title, Abstract, Keyword, English Language, Peer-review |
| AND | “Respectful relationship*” OR “responsive relationship*” | Title, Abstract, Keyword, English Language, Peer-review |
| AND | Collaborat* OR cooperat* OR teamwork OR partnership OR alliance OR “group effort” OR “group work” OR leadership OR “conflict resol*” OR communicat* OR “accepting differences” | Title, Abstract, Keyword, English Language, Peer-review |
| AND | Ethic* OR moral* OR fairness OR "fair play" OR integrity OR equity OR respect OR virtue OR "moral goodness"  OR "moral education" OR "moral value*" OR morality OR "moral obligation*" OR "moral understanding" OR "moral action" OR "moral principles" OR "moral character" | Title, Abstract, Keyword, English Language, Peer-review |
| AND | Society OR divers* OR culture OR “cultur* value*” OR “cultur* divers*” | Title, Abstract, Keyword, English Language, Peer-review |
| AND | "Content knowledge" OR "knowledge level" OR knowledge OR "factual knowledge" | Title, Abstract, Keyword, English Language, Peer-review |
| AND | Safety OR risk* OR endanger* OR “risk manage*” | Title, Abstract, Keyword, English Language, Peer-review |
| AND | Rule* OR regulation* OR guideline* OR principle* | Title, Abstract, Keyword, English Language, Peer-review |
| AND | Reasoning OR “logic* N2 think*” | Title, Abstract, Keyword, English Language, Peer-review |
| AND | Strateg* OR plan* OR approach* OR “line of attack” | Title, Abstract, Keyword, English Language, Peer-review |
| AND | Tactic* OR "tactical knowledge" OR "tactical skills" OR "tactical approach*" OR "tactical efficiency" OR "tactical awareness" OR “tactical manoeuvre*” | Title, Abstract, Keyword, English Language, Peer-review |
| AND | Awareness OR "spatial aware*" OR "spatial perception" OR "spatial reasoning" OR “percept* aware*” OR “sense of awareness” | Title, Abstract, Keyword, English Language, Peer-review |
| AND | “Physical literacy” OR “phy* lit*” | Title, Abstract, Keyword, English Language, Peer-review |
