# Supplementary material for: A systematic review of tools designed for teacher proxy-report of children’s physical literacy or constituting elements
Source: Int J Behav Nutr Phys Act. 2021 Oct 8;18:131. doi: 10.1186/s12966-021-01162-3 (PMC8499583; doi:10.1186/s12966-021-01162-3)
Supplement: Supplementary file 3 — Additional file 3. PICO-based (Population, Intervention, Comparison, Outcome) taxonomy of reasons used to exclude articles from the systematic review. [file 12966_2021_1162_MOESM3_ESM.docx]

PICO-based (Population, Intervention, Comparison, Outcome) taxonomy of reasons used to exclude articles from the systematic review

| **PICO Category** | **Taxonomy code** | **Description** | **Description tag on Covidence** |
| --- | --- | --- | --- |
| Population (P) | Participants with disability | Studies utilizing participants with disability were excluded from the review (exception of those with DCD). | - Participants with disability - Special population - Wrong patient population |
|  | Wrong age group | Participants were not in the desired age range (5-12 years). Since the desired population was children (aged 5-12 years), studies were excluded if they enrolled adults. | - Not primary aged - Adult population |
|  |  | Studies were excluded if they enrolled participants in pre-school or infant/paediatric population. | - Preschool population - Infant population |
| Intervention (I) | N/A | N/A | N/A |
| Comparison (C) | Wrong tool type/study design | The design of the study/tool was not the one desired. | - Self-assessments - Peer report - Parent report - Wrong instrument type (i.e. not proxy-report) - Not focused on assessment of psychometric properties |
|  | Review/Thesis/Conference abstracts/Protocol paper | Review papers were excluded from the systematic review. | - Review paper - Thesis - Conference abstract |
| Outcome (O) | Wrong outcome | The study used the wrong outcome | - Tool not relevant to physical literacy - Wrong construct assessed - Wrong outcomes |
| Not a PICO based exclusion criteria (N) | Article not available | Authors involved in full-text screening of the systematic review were unable to obtain the full text of the article. | - Full-text article cannot be accessed |
|  | Wrong language | The study was published in a language not accessible to the reviewers. | - Study not in English Language |
